# Regulation of TRIB1 abundance in hepatocyte models in response to proteasome inhibition

**DOI:** 10.1038/s41598-023-36512-7

**Published:** 2023-06-08

**Authors:** Sébastien Soubeyrand, Paulina Lau, Ruth McPherson

**Affiliations:** 1 Atherogenomics Laboratory, University of Ottawa Heart Institute, Ottawa, Canada; 2 Division of Cardiology, Ruddy Canadian Cardiovascular Genetics Centre, University of Ottawa Heart Institute, Ottawa, Canada

**Keywords:** Biochemistry, Cancer, Cell biology

## Abstract

Tribbles related homolog 1 (TRIB1) contributes to lipid and glucose homeostasis by facilitating the degradation of cognate cargos by the proteasome. In view of the key metabolic role of TRIB1 and the impact of proteasome inhibition on hepatic function, we continue our exploration of TRIB1 regulation in two commonly used human hepatocyte models, transformed cell lines HuH-7 and HepG2. In both models, proteasome inhibitors potently upregulated both endogenous and recombinant TRIB1 mRNA and protein levels. Increased transcript abundance was unaffected by MAPK inhibitors while ER stress was a weaker inducer. Suppressing proteasome function via *PSMB3* silencing was sufficient to increase *TRIB1* mRNA expression. ATF3 was required to sustain basal TRIB1 expression and support maximal induction. Despite increasing TRIB1 protein abundance and stabilizing bulk ubiquitylation, proteasome inhibition delayed but did not prevent TRIB1 loss upon translation block. Immunoprecipitation experiments indicated that TRIB1 was not ubiquitylated in response to proteasome inhibition. A control *bona fide* proteasome substrate revealed that high doses of proteasome inhibitors resulted in incomplete proteasome inhibition. Cytoplasm retained TRIB1 was unstable, suggesting that TRIB1 lability is regulated prior to its nuclear import. N-terminal deletion and substitutions were insufficient to stabilize TRIB1. These findings identify transcriptional regulation as a prominent mechanism increasing TRIB1 abundance in transformed hepatocyte cell lines in response to proteasome inhibition and provide evidence of an inhibitor resistant proteasome activity responsible for TRIB1 degradation.

## Introduction

The *Tribbles* family comprises 3 evolutionary divergent pseudokinases with roles in lipid metabolism, MAPK signaling, cell proliferation and cancer^1^. Mammalian *tribbles* interact with several proteins, including the E3 ligase COnstitutively Photomorphogenic 1 (COP1) and CCAAT/enhancer-binding proteins (CEBPs)^2,3^. By bringing CEBPA to COP1, TRIB1 and TRIB2 facilitate CEPBA degradation thereby controlling the lipogenic potential of this key transcription factor^4,5^. In humans, genetic variants proximal to *TRIB1* have been associated with non-alcoholic fatty liver disease^6,7^. In mice, liver-specific TRIB1 deletion results in the hepatic accumulation of lipids and steatohepatitis-like features^4^. The fate of TRIB1 as an adaptor protein is unclear. E3 adaptor proteins, including TRIB1, tend to be unstable^8^. For TRIB3, COP1 overexpression reduces CEBPA without affecting TRIB3 levels, suggesting that it is not degraded via COP1^9^. Interestingly, the same *Tribbles* Val-Pro interface mediates binding of both adaptor proteins and degradation targets to COP1^10,11^.

Regulated protein degradation involves three major pathways. Removal of ubiquitylated proteins typically occurs via the 26S, a large protein complex exhibiting 3 distinct protease activities^12–14^. Several proteasome inhibitors (PIs) have been developed over the years, some of which have clinical implications^15,16^. Chief among them is Velcade/Bortezomib (BTZ), used in the treatment of multiple myeloma. The impact of proteasome inhibitors on liver disease is still unresolved. On the one hand, BTZ therapy is sporadically linked to liver injury and has been shown to exhibit hepatotoxicity in mouse models^17^. On the other hand, proteasome inhibition may also be protective by reducing liver fibrosis and increasing anti-oxidative defenses^18–20^. In addition to proteasome-mediated degradation, lysosomes and autophagosomes also contribute to the removal of larger cargos via lysosomal proteases^13,21^. All 3 pathways share some dependence on ubiquitylation and are likely functionally interconnected, particularly under stress^22,23^. Finally, additional proteolytic pathways, sometimes detectable only in the absence of functional proteasome, have been described, including Calpains, TPP2 and others^24–28^.

We previously demonstrated that TRIB1 regulation in non-liver cell lines occurred at multiple levels, involving proteasome dependent and independent processes at transcriptional and post-transcriptional levels^29^. An intriguing finding was that whereas drug mediated proteasome inhibition was sufficient to increase TRIB1 levels, associated with increased transcription, proteasome inhibition was insufficient to prevent loss of TRIB1 after CHX treatment, hinting to alternate pathways that could degrade TRIB1. The current study explores the regulatory processes controlling TRIB1 abundance in greater depth, leveraging commonly used hepatocyte model cell lines, HepG2 and HuH-7, of hepatoblastoma and hepatocarcinoma origin respectively. Our results indicate that proteasome inhibition augments TRIB1 abundance through direct and indirect mechanisms, by hindering proteasome mediated TRIB1 degradation as well as through transcriptional upregulation.

## Materials and methods

### Cell culture and treatments

HuH-7 (Japanese Collection of Research Bioresources Cell Bank) and HepG2 (ATCC) were maintained in physiological (5 mM) glucose DMEM media supplemented to 10% FBS and containing Antibiotic–Antimycotic (Gibco). Drugs are listed in the “Supplementary Materials and Methods” section. Stable cell pools were obtained by 3 µg/ml puromycin selection following viral transduction. Vehicle (DMSO or PBS) was added at 1% or less, final concentration, as indicated. For siRNA cells (24 well plate, 0.5 ml per well of culture media) were transfected at 30% confluence for 72 h (TRIB1) or 96 h (PSMB3) with the addition of RNA iMAX (ThermoFisher;1 µl) and a TRIB1 siRNA (6 pmol) in 100 µl of Optimem (Gibco). siRNAs are listed in the “Supplemental material” section. Unless mentioned otherwise, drug treatments were combined and continuous. For pulse-chase set (Fig. 6), cells were treated for 2 h with BTZ followed by 2 media rinses and a 5 h wash-out period in the presence or absence of CHX (10 µg/ml).

### Western blotting

For Western blot analyses, protein lysates were obtained by lysing cells in cell lysis buffer (PBS supplemented with 1% Triton X-100 as well as protease (EDTA-free Complete, Roche) and protein inhibitor cocktails (PhoSTOP, Roche)) for 5 min on ice. Lysates were then cleared by centrifugation (13,000 xg for 2 min @ 4 C). Approximately 30 µg per lane as assessed using a Bradford assay (Coomassie Reagent, Bio-Rad) were heat denatured at 95 °C under reducing conditions in Laemmli sample buffer (5 min) and resolved on 1.5 mm 8% SDS-PAGE gels unless mentioned otherwise. Proteins were transferred to nitrocellulose membranes using semi-dry transfer (Trans-Blot Turbo, Bio-Rad) for 6 min at 2.5 A or liquid transfer (90 V, 60 min), with similar results. Membranes were routinely Ponceau-stained (0.1% Ponceau S in 1% acetic acid) prior to blocking to ascertain transfer quality. Blots were fully then destained in PBS and blocked in Intercept buffer (LI-COR, 30 min) and rinsed briefly in PBS/0.1% Tween (PBS/T). Incubation with the primary was typically performed in PBS/T for 16 h. Secondary antibodies were from Li-Cor (680 and 800) and were used as 1:20,000 dilutions. Antibody incubations were followed by one PBS/T and three PBS rinses. Western blots were imaged on an Odyssey Infrared Imaging station and quantified using Image Studio Lite (Li-Cor), using local Median background values. Sample values were internally normalized to protein load using Tubulin Beta (TUBB) values. Values were then expressed relative to the corresponding vehicle-treated control unless mentioned otherwise. All antibodies used are listed in the “Supplemental material” section.

TRIB1 is expressed at very low levels and was undetectable in naïve HuH-7 and HepG2 using 2 distinct Abs under basal conditions; lack of TRIB1 signal was ascertained using orthogonal validation using antisense oligonucleotides and lack of responsiveness to actinomycin D (^30^ and data not shown). Different lots of TRIB1 antibodies raised in rabbits (TRIB1r; targeting the N-terminal region) were inconsistent in sensitivity but yielded comparable background signals; unfortunately, more recent lots of this antibodies had unacceptable background and could not be used. The goat-raised TRIB1 (TRIB1g, targeting the C-terminal region) was more consistent across lots but exhibited slightly lower sensitivity. Analyses were performed with either or both Ab as limited supplies permitted, as indicated. Note that both Abs revealed the presence of an overlapping non-specific band (resistant to TRIB1 siRNA and ACTD), noticeable mainly with longer exposures (i.e., for the endogenous, low abundance, protein). Exogenous wild-type TRIB1 was readily detectable as a tight band triplet with a prominent central band, using either antibody.

### Immunoprecipitation

Whole cell lysates of HepG2-T1 (stably transduced with pLVXpuro-TRIB1) cells were obtained by lysing cells in PBS containing 0.1% Triton X-100 (PBS/TX) as well as protease (EDTA-free Complete, Roche) and protein inhibitor cocktails (PhoSTOP, Roche) for 5 min on ice. For native immunoprecipitations, cleared lysates (13,000 xg for 2 min @ 4 C) were adjusted to 1 mg/ml with PBS/TX buffer and antibodies were added at 2 µg/mg lysate for 30 min, followed by the addition of protein A/G beads (10 µl suspension; PureProteome, EMD Millipore) for an additional 5 h on an orbital plate. Immunoprecipitates were then washed 4X with 0.5 ml of PBS/TX buffer, heat denatured at 95 °C under reducing conditions in Laemmli buffer (5 min) and analyzed by Western blot.

For immunoprecipitation of denatured samples, a 10 cm dish of 70% confluent HepG2-T1 was transfected for 48 h with 5 µg of HA-Ubiquitin plasmid (Addgene plasmid # 18,712; a gift from Edward Yeh^30^) with X-tremeGENE HP at a ratio of 1:3 (DNA to reagent). Denaturation and immunoprecipitation were then performed essentially as described previously^31^. Briefly, cleared lysates (0.5 mg) were adjusted to 1% SDS, heated at 95 °C and diluted in a 1% Triton X-100 containing buffer prior to immunoprecipitation with 2 µg of either a rabbit antibody to TRIB1 (N2C3, GeneTex) or a rabbit mCherry antibody (NBP2-25,157, Novus Biologicals). Immunoprecipitation was performed for 16 h in the presence of protein A/G beads and bead bound material was washed and processed as described above.

### Real-Time RNA quantification (RT-qPCR)

RNA was extracted from culture plates using Tri Reagent (Roche) and isolated using Direct-Zol RNA miniprep kits (Zymo Research). RNA (0.5 µg) was reverse transcribed using the Transcriptor First Strand cDNA kit (Roche), using a 1:1 mixture of oligo dT and random hexamer primers for 1 h. Resulting cDNA was diluted fivefold in H_2_O prior and quantified on a Light Cycler 480 using SYBR Green (Roche), using 0.5 µM of primers. Target of interest values were expressed relative to the corresponding peptdidyl Peptidylprolyl Isomerase A (PPIA) values using the 2 − ΔΔCt method^32^. Oligonucleotides are listed in the “Supplemental material” section.

### Plasmid and lentiviral constructs

TRIB1NES, wherein the bipartite TRIB1 nuclear localization sequence (AA 31–51) was substituted for a PKIA nuclear export signal as described previously, was subcloned into PLVX-puro^33^. For the PEST substituted construct, a synthetic fragment containing the GST helical region, consisting of 2 identical helices, was used to replace the PEST region (AA 53–88) of TRIB1 (see “Supplemental material” section for sequence). N-terminal truncated TRIB1 expression constructs (Δ2-51 and Δ2-91) were obtained by sub-cloning TRIB1 from the corresponding pCFP-TRIB1 construct to pLVX via BamHI transfer. pJA317 and pJA291 were a gift from Andrew Fire obtained from Addgene^34^. Both plasmids express GFP, with (pJA317) or without (pJA291) the ODC1 degron, separated from a puromycin-N-acetyltransferase-mCherry fusion by a T2A ribosome skipping site. Constructs were packaged in 293FT cells using co-transfection of pLVX constructs with psPAX2 and pMD2.G (Addgene). Virus titers were estimated using puromycin resistance in HEK-293 T cells; an equivalent of 3 Multiplicity of Infections (MOIs), defined as 3 times the amount of virus sufficient to render HEK293T resistant to 2 µg/ml puromycin, was used per infection.

### Microscopy

Cells were seeded on glass coverslips and fixed with 4% PFA in PBS for 15 min and subsequently permeabilized and blocked for 20 min in PBS containing 0.1% Triton X-100 and 5% FBS. Detection was performed by incubating with primary antibodies at 1:500 dilutions in PBS and secondaries (Jackson laboratories, Alexa633 coupled donkey anti-goat and Alex488 coupled anti-rabbit) at 1:2000 for 1 h in PBS/0.1% Triton. Four 1-min rinses in PBS were performed after each incubation. Cells were then mounted in Fluorescent Mounting Medium (DakoCytomation) supplemented with 1 µM Hoechst 33,342. Microscopy was performed using a 63 X Oil Immersion lens on a Zeiss Elyra microscope using the Zeiss Zen software. Images were acquired in 2 or 3 channels using sequential excitation/emission. In images shown only linear changes in intensity and contrast were performed (K = 0).

### Statistics

Statistical significance between groups was tested with either one-way ANOVA or pairwise comparisons with a Student’s *t* test, as indicated in the figure legends. Error bars represent standard deviations throughout the manuscript.

## Results

### TRIB1 abundance in hepatocyte cell lines is upregulated by proteasome inhibitors

**Figure 1:**
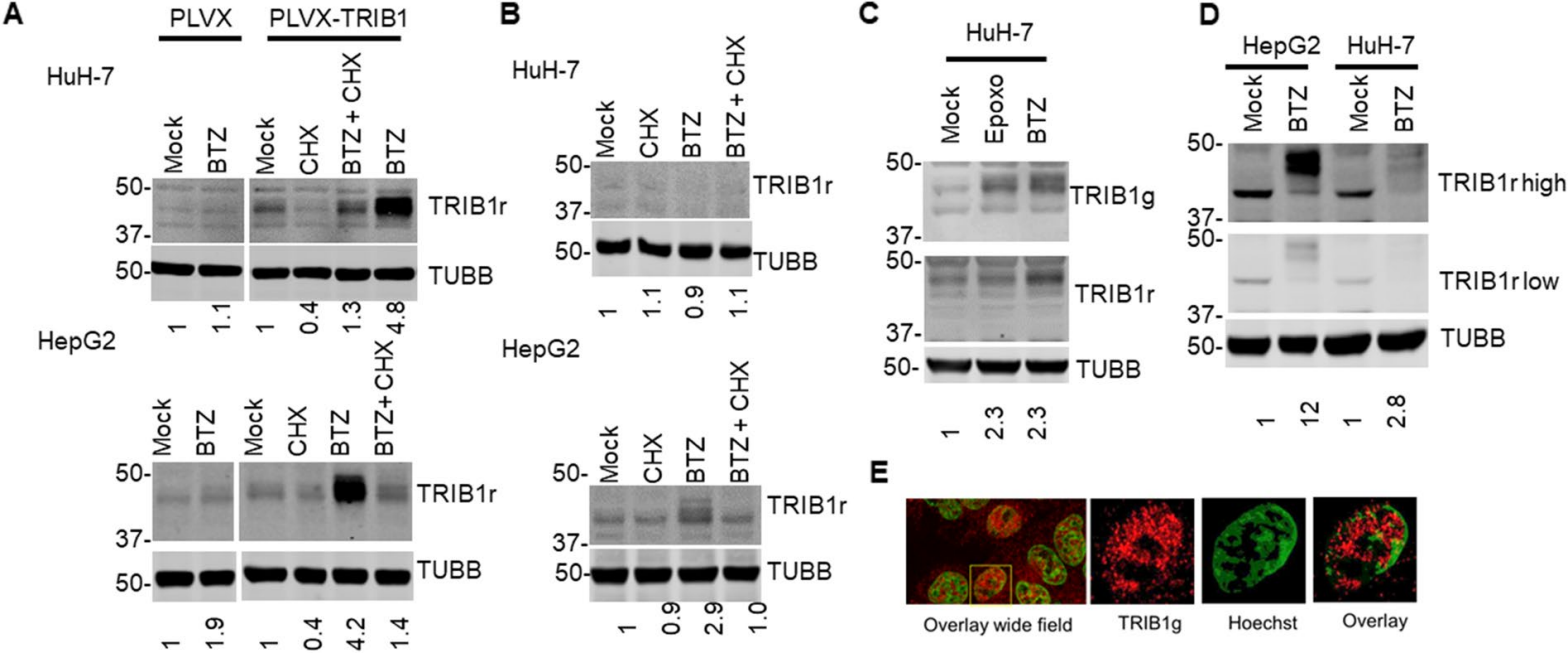
Sensitivity of over expressed and endogenous TRIB1 in HuH-7 and HepG2 cell lines to BTZ and CHX. Western blot analyses of transduced (**A**) or untransduced (**B**–**D**) HuH-7 and HepG2 cells treated with proteasome inhibitors and/or CHX. Detection was performed either with goat (TRIB1g) or rabbit (TRIB1r) raised Abs (in distinct channels), as indicated. A, PLVX and PLVX-TRIB1 stably transduced cells were exposed to a 5 h BTZ treatment in presence of CHX or vehicle (1% DMSO). Cropped regions from the same blots are shown. (**B**) as in (**A**) but parental (i.e., uninfected) HepG2 and HuH-7 were treated as indicated. Data representative of 3 experiments. (**C**) TRIB1 signal in HuH-7 is detectable in response to a prolonged (18 h) treatment with 2 µM epoxomicin (Epoxo) or 5 µM BTZ. (**D**) Relative abundance of TRIB1 in HepG2 and HuH-7 cells following an 18 h incubation with BTZ. Two different exposures (high or low) are shown. Data are representative of 3–5 experiments. Quantification is expressed relative to TUBB and normalized to the Mock sample; for TRIB1 quantification, the TRIB1r signal was used. (**E**) HepG2 cells seeded on coverslips were treated with BTZ (5 µM) for 5 h. Detection was performed using a TRIB1 Ab followed by an Alexa633 coupled donkey anti-goat (TRIB1g; red). Nuclei were stained with 1 µM Hoechst (green) signal. Left, representative overlay of signals from a group of cells. Right, individual channels of highlighted nucleus. Image was smoothed with a Gaussian filter (default parameter settings ZEN 3.3). The experiment was repeated 3 times with similar results.

In liver cell lines, the PI bortezomib (BTZ) increased recombinant TRIB1, detectable as 2–3 closely migrating bands centered around a prominent ~ 45 kDa, as observed previously in transduced non-liver cell models and unexpectedly revealed a weak TRIB1 signal in vector transduced HepG2 (Fig. 1A)^33^. The presence of multiple novel bands was confirmed in naïve HepG2 but not in HuH-7 treated for 5 h with BTZ (Fig. 1B). Moreover, as previously observed in non-liver cell lines, TRIB1 was unstable, as revealed by cycloheximide (CHX) treatment, resulting in the reduction (recombinant TRIB1) or disappearance (endogenous TRIB1) of the BTZ-dependent TRIB1 signal within 5 h of the translation inhibitor addition (Fig. 1A,B). Increased TRIB1 was accompanied by a 3 to 14-fold *TRIB1* transcript increase in HepG2 and HuH-7 cells, respectively and was observed with other, mechanistically, and structurally distinct, inhibitors Epoxomycine (EPOXO) and Lactacystin (LACTA) (Figure S1). With prolonged BTZ incubation, the TRIB1 signal became more intense in HepG2 and detectable in HuH-7 cells, indicating that TRIB1 accumulates over time (Fig. 1C,D). In addition, the endogenous signal observed after BTZ incubation was reduced in TRIB1-silenced cells, verifying its identity as TRIB1 (Figure S2). Immunofluorescence revealed a faint TRIB1 antibody reactive signal that was enriched in the nucleus following BTZ treatment, sensitive to CHX, and detectable as early as 5 h in HepG2 cells (Fig. 1E, Figure S3). This is consistent with our prior work demonstrating that recombinant TRIB1 is a nuclear resident in several model systems, including HepG2 cells^31,33^. Although a weak nuclear signal could be observed in HuH-7 after a 16 h BTZ treatment, it was insensitive to a 5 h CHX regimen, hinting at a possible off-target signal (data not shown).

### Increased TRIB1 can be blocked by transcription inhibitor actinomycin D

**Figure 2:**
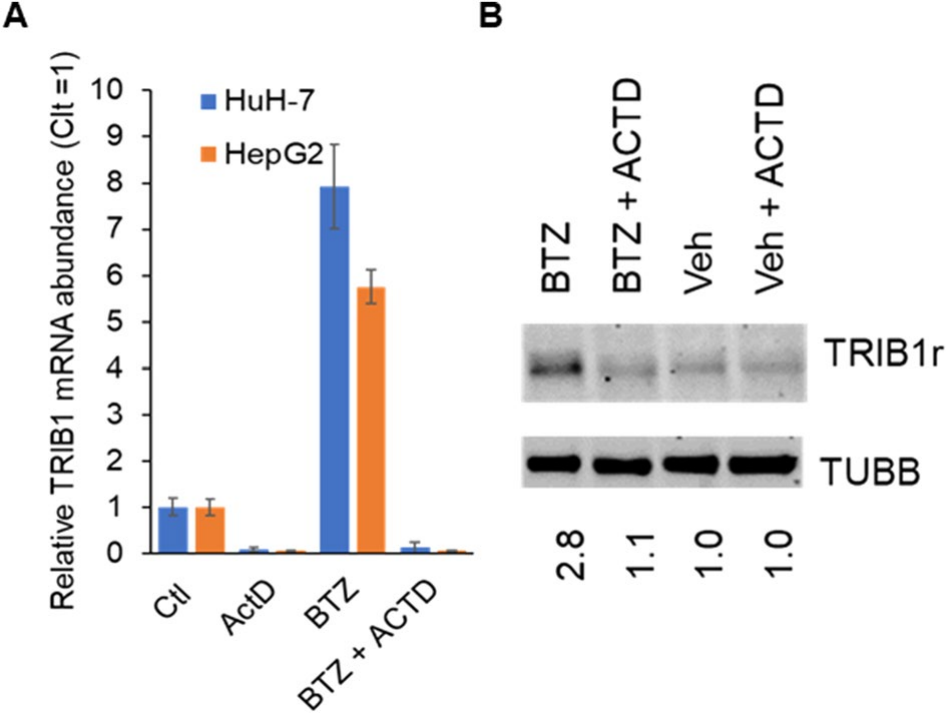
Transcription inhibition with actinomycin D prevents endogenous TRIB1 upregulation. (**A**, **B**) HuH-7 and HepG2 cells were treated with BTZ or vehicle (Veh; 1% DMSO), with and without (Ctl) a preincubation with actinomycin D (ACTD) for 5 min. (**A**) mRNA abundance in HuH-7 and HepG2 cells. Bars are the average of 3 biologics (± SD). Within each cell type, differences between Ctl and ACTD were highly significant (p < 0.001, one-way repeated measures ANOVA). (**B**) Western blot performed in HepG2 cells treated as in A; endogenous protein signal was undetectable in HuH-7 after 5 h BTZ. Quantifications represent TUBB normalized signal intensity, expressed relative to the corresponding Ctl values. Data representative of 3 experiments.

The role of transcription in regulating TRIB1 abundance in response to BTZ was examined by blocking de novo POLII transcription with ACTD. Comparable fold reductions in *TRIB1* mRNA were observed upon treating control and BTZ treated cells with ACTD, suggesting that ongoing transcription is required (Fig. 2A). Moreover, ACTD treatment was sufficient to prevent BTZ mediated protein accumulation (Fig. 2B). Thus, transcription plays a determining role in driving TRIB1 protein accumulation.

### Increased *TRIB1* mRNA abundance in response to proteasome inhibitors is unaffected by MAPK inhibitors

MAP kinases (p38, JNK and ERK1/2) are major regulators of cell proliferation and stress response and may contribute to increased TRIB1 transcription in response to BTZ induced proteotoxic stress. Using inhibitor based approaches, we previously demonstrated the essential role of ERK1/2 in promoting TRIB1 transcription following mitochondrial stress in HepG2 cells^35^. The contribution of ERK1/2 was tested by blocking ERK1/2 activity with PD98059; this however had no effect on the BTZ mediated increase in TRIB1 (Figure S4). Proteasome inhibition also stabilizes and activates c-Jun via JNK; Jun is a major component of AP-1 complex, which occupies the TRIB1 promoter in naïve HepG2 cells and liver according to ENCODE data^36–38^. As for p38, it can be activated by proteasome inhibition and has the potential to promote AP-1 function^39,40^. As observed for ERK inhibition, inclusion of JNK and p38 inhibitors failed to block the BTZ mediated increase. Finally, combining all 3 had minimal impact on TRIB1 upregulation by BTZ, implying that increased *TRIB1* mRNA abundance does not require the contribution of MAPKs.

### BTZ induces changes consistent with the establishment of a moderate ER stress response

**Figure 3:**
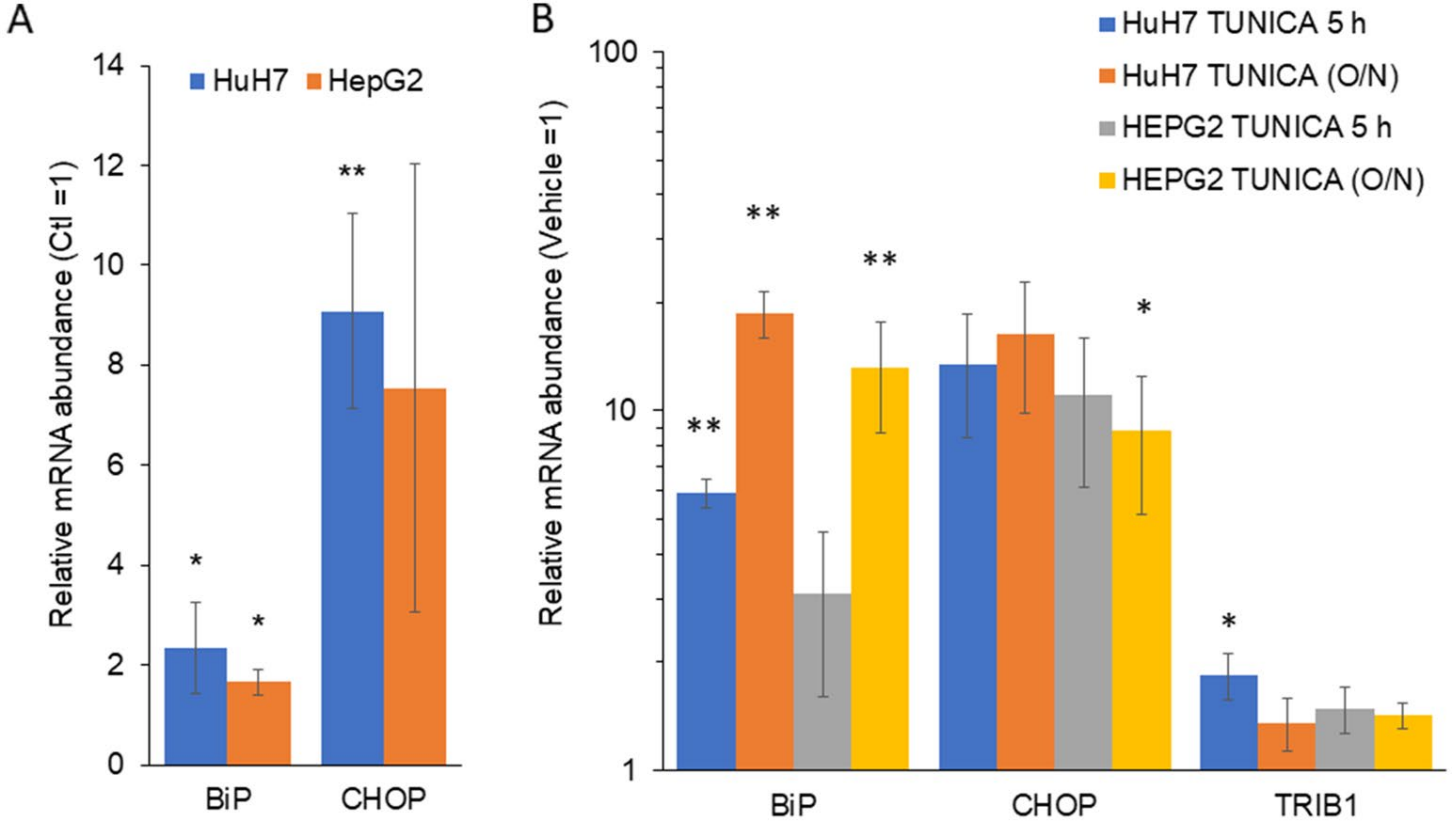
BTZ induces changes consistent with the establishment of an ER stress response. Cells were treated with (**A**) BTZ (5 µM, 5 h) or (**B**) Tunicamycin (12 µM) for either 5 or 18 h (O/N). RNA was then isolated and quantified by qRT-PCR. Bars represent the means of 3 biological replicates ± S.D. Values were first normalized to PPIA (ΔΔCT method) and are expressed relative to vehicle (1% DMSO) values. Bars represent the means of 3 biological replicates (± S.D.). Paired Student *t* test, BTZ versus vehicle. *P < 0.05; **P < 0.01 from vehicle.

Proteasome inhibition, by interfering with protein turnover of misfolded proteins targeted for secretion, has the potential to induce ER stress and an unfolded protein response. Interestingly TRIB3 has been shown to be highly responsive to ER stress induction via tunicamycin in HepG2 cells^41^. Moreover, liver-specific *Trib1* deletion leads to increased EIF2a phosphorylation, a marker of ER stress, suggesting that TRIB1 may contribute to ER function^5^. BTZ treatment elicited a stress response as assessed by increased CHOP and BiP in both cell lines (Fig. 3). To examine the potential of ER stress in controlling TRIB1 levels, cells were treated with tunicamycin, a prototypical ER stress inducer. A 5 h (or 22 h) tunicamycin modestly increased TRIB1 abundance (1.1–twofold); by comparison, tunicamycin treatment resulted in 5 to 20-fold BiP and CHOP increases in both cell lines (Fig. 3). Thus, while BTZ induces an ER stress these cell lines, it may be insufficient to account for the 4 to 15-fold increased TRIB1 mRNA levels elicited by PI treatments in HepG2 and HuH-7, respectively.

### Regulation of TRIB1 expression by ATF3

To initiate our search for possible mediators responsible for the PI dependent upregulation, transcription factors upregulated in response to PI treatment were examined. Specifically, published data which measured the impact of BTZ on HepG2 cells transcriptome by RNAseq, were interrogated (GSE166923 at the Gene Expression Omnibus). Genes that were upregulated by more than twofold by a 10 h 50 nM BTZ regimen (757 genes out of 18,915, including TRIB1 (3.5-fold)) were subjected to over-representation analysis (ChIP-X enrichment analysis; ChEA) with Enrichr (https://maayanlab.cloud/Enrichr/)^42^. The most enriched TF was ATF3, a stress response mediator whose mRNA abundance was strongly increased (~ 26-fold) in BTZ-treated HepG2s, and which interacts with the *TRIB1* gene promoter region in (naïve) HepG2 cells (Figure S5A,B). Furthermore, ATF3 was upregulated in livers of *Trib1* deleted mice, consistent with a functional interplay^5^. To test the contribution of ATF3 to *TRIB1* expression, ATF3 was silenced for 72 h in HepG2 and HuH-7 cells. Singly, *ATF3* suppression (by 59% in HuH-7 and 68% in HepG2) reduced TRIB1 modestly (by 36% in HuH-7 and 23% in HepG2) consistent with ATF3 contributing to basal TRIB1 expression (Figure S5C). Upon BTZ addition, *ATF3* silencing resulted in a reduced response in HuH-7 cells and a similar trend in HepG2 cells (Figure S5C). The impact of ATF3 suppression on *TRIB1* abundance in BTZ treated cells approached the corresponding basal values (i.e., reductions of 42% and 20% vs [NT1si + BTZ] in HuH-7 and HepG2 respectively), suggesting ATF3 contributes similarly to basal and BTZ induced *TRIB1* expression.

### Rapid upregulation of TRIB1 by proteasome inhibitors

**Figure 4:**
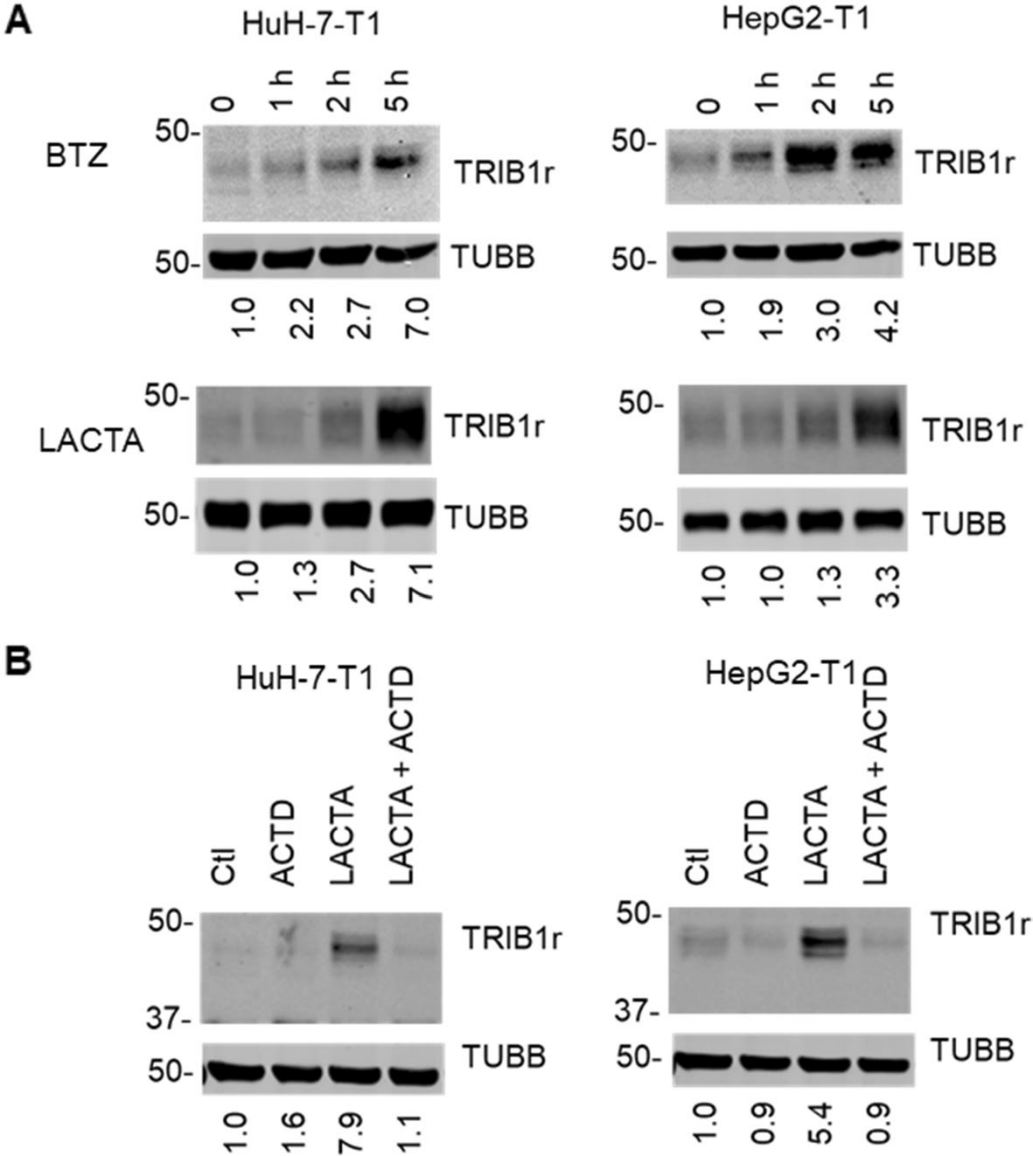
Time course of proteasome inhibitors on TRIB1 overexpressing cells. (**A**) Stable pools of HuH-7-T1 and HepG2-T1 cells were treated for 1 to 5 h with BTZ (5 µM) or LACTA (10 µM) for the indicated time. (**B**) HuH-7-T1 and HepG2-T1 cells were incubated for 5 h with lactacystin (LACTA,10 µM) and/or Actinomycin D (ACTD, 5 µg/ml), as indicated. Samples were resolved by Western blot and analyzed with the indicated antibodies. Quantifications represent TUBB normalized signal intensity, expressed relative to the corresponding Ctl values. Data representative of 3 experiments.

A time course was performed to better characterize the upregulation at the protein level. To visualize TRIB1 at early time points, when endogenous protein abundance is too low for detection, the assay was performed on recombinant TRIB1. To address possible off-target BTZ effects on non-proteasome proteases^43^, lactacystin (LACTA), a structurally and mechanistically distinct inhibitor was also used^15^. Lactacystin was included at a concentration previously shown to inhibit ~ 70% of the proteasome chymotryptic activity in situ^44^. Using BTZ or LACTA, rapid TRIB1 upregulation was detectable within 1 h of treatment and increased over 5 h (Fig. 4A). Transduced wild-type TRIB1 was readily detectable as multiple closely migrating bands (typically a tight triplet with a prominent central band), using either antibody, as observed previously in other cell models^33^. As observed above with the endogenous transcript, PIs potently upregulated recombinant *TRIB1* mRNA (Figure S6). As noted earlier with the endogenous protein, upregulation was blocked by ACTD indicating that endogenous and recombinant TRIB1 protein abundance both require transcription (Fig. 4B).

### Proteasome activity suppression via *PSMB3* silencing increases total ubiquitylation and *TRIB1* mRNA abundance

Evidence to date points to the major contribution of transcription in regulating TRIB1 abundance when the proteasome is inhibited. These experiments, however, were performed in the presence of inhibitors with known off-target effects. To address these limitations, the proteasome core beta 3 subunit (PSMB3) was suppressed by siRNA **(**Figure S7). Impaired proteasome function was ascertained by the increased abundance of coupled ubiquitin chains. The incubation, which showed no sign of obvert toxicity by microscopic inspection, was however insufficient to reveal endogenous TRIB1 protein signal, despite increasing *TRIB1* mRNA (2.8-fold and 8.2-fold in HepG2 and HuH-7 cells, respectively; significant only in HepG2) (Figure S7A). Lack of detection is consistent with no or low protein upregulation. To distinguish between these 2 scenarios, proteasome inhibition via PSMB3 was repeated on cells expressing the ~ 10–20-fold more abundant recombinant *TRIB1*. Reduced *PSMB3* levels were associated with a modest increase in TRIB1 protein abundance, which reached statistical significance only in HepG2-T1 (1.2-fold ± 0.07; n = 3, *p* < 0.05) and could be due to a correlative 1–5-twofold increased *TRIB1* mRNA (Figure S7B). Thus, compromised proteasome activity via *PSMB3* suppression was sufficient to increase *TRIB1* mRNA but insufficient to phenocopy the impact of PIs.

### TRIB1 loss upon CHX occurs more rapidly than bulk ubiquitin

**Figure 5:**
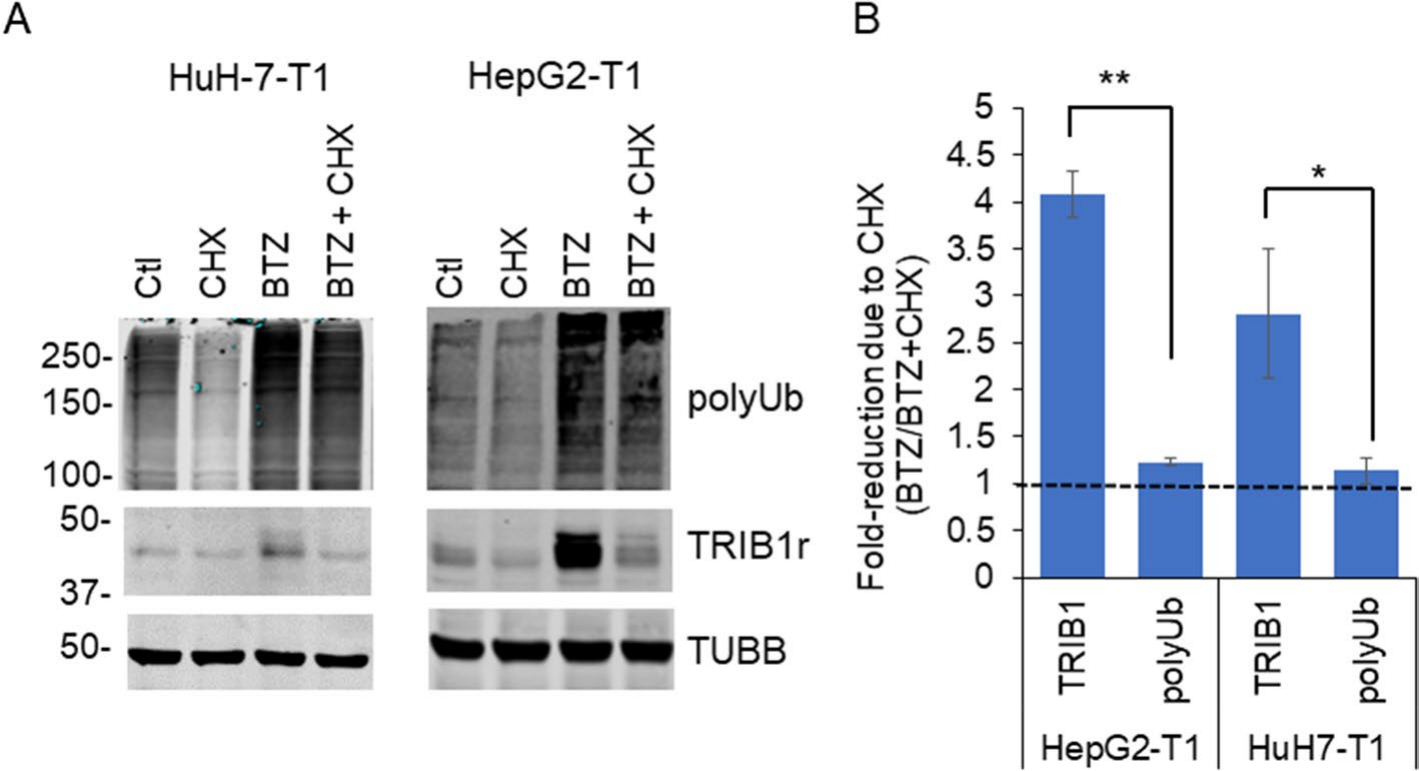
In the presence of BTZ, loss of polyUb in response to CHX is slower compared to TRIB1. Western blot analysis of HuH7-T1 and HepG2-T1 treated with the indicated drugs for 5 h. (**A**) Representative Western blots. Levels of polyUb were quantified over the 100–150 kDa range. (**B**) Relative sensitivities of TRIB1 and polyUb signals to BTZ and CHX after a 5 h treatment. Quantifications of the TRIB1 signals and polyUb (TUBB corrected) in HepG2-T1 and HuH-7-T1 cells following BTZ and/or CHX treatments. Dashed line indicates a no-loss scenario. Average of 3 independent experiments. *p < 0.05; **p < 0.01; Unpaired *T* test, TRIB1 versus polyUb.

Having explored mechanisms driving increased TRIB1 expression, we turned our attention to the processes responsible for TRIB1 protein instability. First, loss of TRIB1 and bulk ubiquitylation in the presence of BTZ and CHX were compared. Loss of coupled Ub was minimal over 5 h (~ 23% and ~ 13% signal loss in HepG2-T1 and HuH-7-T1 cells, respectively) consistent with compromised proteasome function (Fig. 5). By contrast, TRIB1 was rapidly degraded under the same conditions, suggesting that the mechanisms responsible for TRIB1 and bulk ubiquitylation removal differ.

### High dose of Carfilzomib or combined PI treatment do not prevent TRIB1 loss to CHX

Preferential loss of TRIB1 versus polyUb could reflect selective targeting of TRIB1 to one of the proteasome proteolytic activities. Since BTZ preferentially targets the chymotrypsin-like and caspase-like activities of the proteasome over the trypsin-like activity, CHX mediated loss of TRIB1 upon PI treatments may reflect incomplete inhibition of the proteasome, possibly with preferential engagement of the tryptic activity. Carfilzomib (CFZ) is an irreversible, clinically relevant, epoxyketone inhibitor that blocks trypsin-like and chymotrypsin-like activities at high doses in situ^45^. As shown, inclusion of CFZ increased polyUb signal had no impact on TRIB1 loss by 5 h (Figure S8A). A similar conclusion was drawn from the combined use of 3 mechanistically distinct inhibitors (BTZ, LACTA and EPOXO) which has been suggested to increase inhibition potency^45^ (Figure S8B).

### TRIB1 loss is delayed by proteasome inhibition

**Figure 6:**
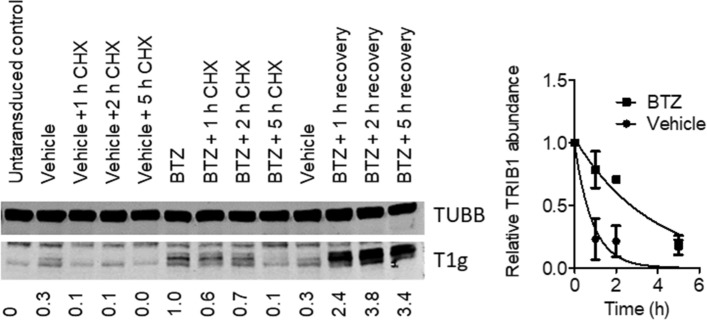
Pulse chase of recombinant TRIB1 in HepG2 cells. Left, representative Western blot of HepG2-T1 cells treated with 5 µM BTZ or vehicle (0.5% DMSO) for 2 h, followed by a wash out period of 0 to 5 h in the presence of CHX (10 µg/ml) or no CHX (recovery). Right, best fit regressions of biological triplicates using the one phase decay model (GraphPad Prism). TRIB1 abundance (TRIB1/TUBB) values were normalized to the matching control value (Vehicle or BTZ). Error bars for the [BTZ, 2 h] time point are contained within the symbol.

Alternatively, residual proteasome activity in the presence of proteasome inhibitors may be insufficient to handle bulk ubiquitylation but sufficient to degrade TRIB1. The impact of BTZ on TRIB1 instability was first examined through a comparative time course approach, comparing TRIB1 instability in the presence and absence of BTZ. HepG2-T1 cells incubated transiently with BTZ or vehicle for 2 h, were allowed to recover for up to 5 h in the presence of CHX. Although excess BTZ was removed from the cell media, CHX-free controls indicated that TRIB1 continued to increase over 2–5 h post wash, presumably due to the strong affinity of BTZ for the proteasome causing persistent proteasome inhibition (Fig. 6). Interestingly, whereas TRIB1 was largely lost after 5 h of CHX treatment, irrespective of BTZ, BTZ pretreatment significantly delayed the disappearance of TRIB1, shifting the apparent half-life from 0.63 h to 2.6 h. (0.39–1.61 to 1.9–3.8 (95% C.I.)). These findings pointed to the contribution of the proteasome in promoting TRIB1 degradation, possibly assisted by other degradation processes.

### Evidence that TRIB1 is not ubiquitylated by overexpression approaches

The above results indicated that the proteasome is instrumental in mediating TRIB1 instability. Ubiquitylation ordinarily precedes degradation by the proteasome although it is not required for some proteins (e.g.^46,47^). Evidence of TRIB1 ubiquitylation, which would further support the implication of proteasome-mediated degradation, was examined by performing immunoprecipitation (IP) under both basal and proteasome inhibited conditions. Proteasome inhibition with BTZ increased total polyUb but failed to reveal a specific TRIB1 associated Ub signal (Figure S9A). To minimize signal loss due to possible in vitro deubiquitylation, IP was repeated on denatured samples. Moreover, cells were transfected with additional ubiquitin (HA-tagged) to promote the formation of possible Ub-TRIB1 conjugates. Similar to the native IP experiments, no Ub-TRIB1 signal could be identified in immunoprecipitates (Figure S9B). Combined with earlier evidence of delayed TRIB1 loss in the presence of PI, these findings were consistent with proteasome-mediated degradation of TRIB1 processing without ubiquitylation.

### PI treatments inhibit but are insufficient to prevent a reporter proteasome substrate degradation in HepG2 cells

**Figure 7:**
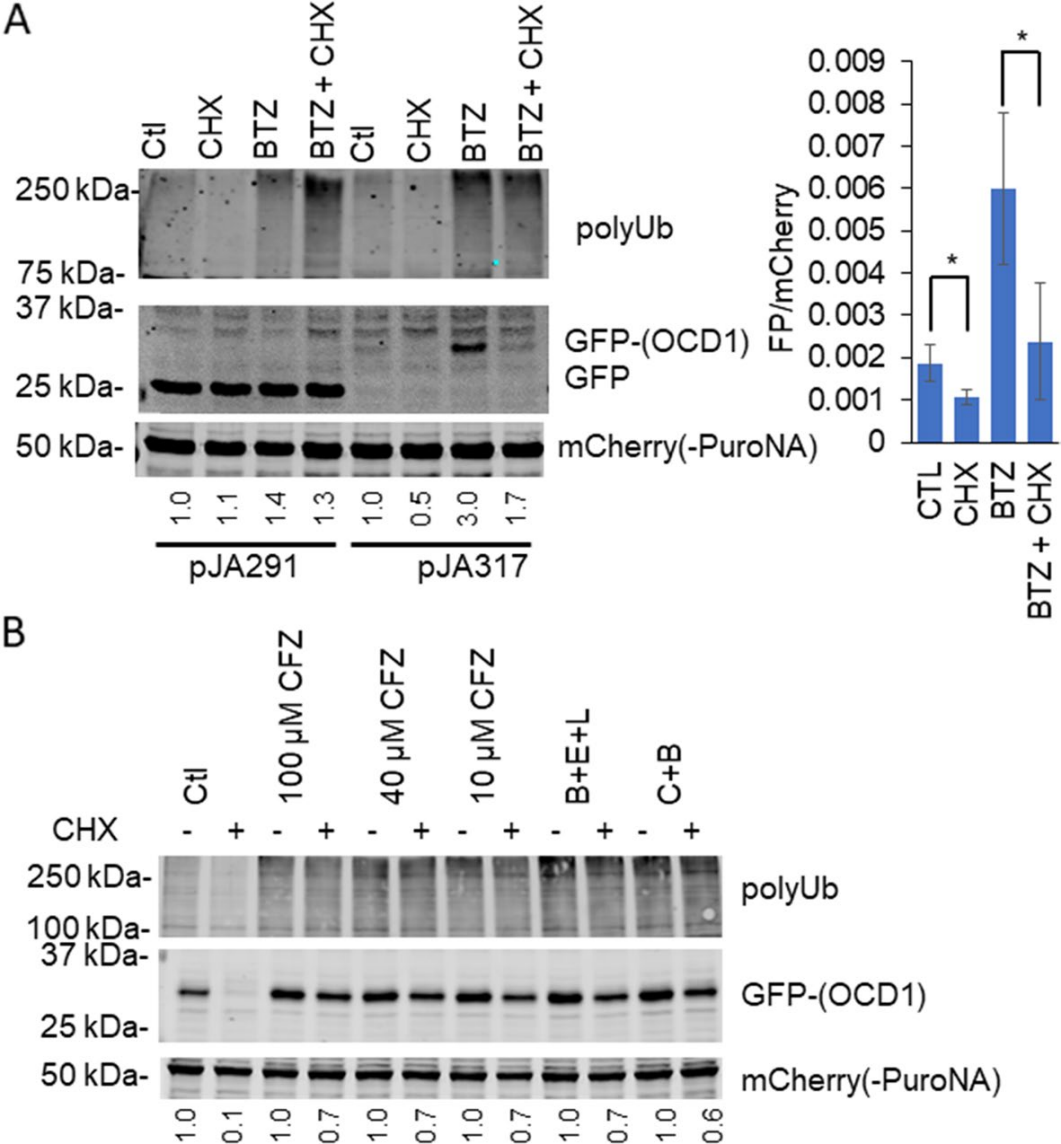
Sensitivity of GFP-OCD1 to CHX in the presence of proteasome inhibitors. Western blot analyses of HepG2 cells stably expressing OCD1 tagged GFP (pJA317) or GFP only (pJA291). (**A**) Left, cells were treated with BTZ or vehicle (0.25% DMSO, Ctl) for 5 h, with or without CHX, as indicated. GFP quantifications are corrected for transduction efficiency using the mCherry signal and normalized to the corresponding control. Right, statistical significance of CHX’s impact was assessed using Student’s paired *t* tests (CTL vs. CHX, BTZ vs. BTZ + CHX) on 3 distinct experiments. (**B**) Carfilzomib dose response and combination experiment. OCD1-GFP expressing HepG2 Cells were treated with the indicated drugs for 5 h, with a 5 min preincubation with PI, as appropriate. B + E + L: Combination of BTZ (B, 5 µM), epoxomicin (E, 2 µM) and lactacystin (L, 10 µM). C + B: combination of BTZ (10 µM) and CFZ (100 µM). mCherry-PuroNA fusion is used as an internal transfection control. Quantifications shown are the GFP signal, divided by the mCherry signal and expressed relative to the matching CHX-free treatment.

Although delayed by PI, significant TRIB1 loss still occurred, which could imply residual proteasome activity or the contribution of additional proteolytic pathways. To assess proteasome status in situ, HepG2 cells were transduced with a recombinant construct consisting of a green fluorescent protein (GFP) fused with a ornithine decarboxylase (ODC) degron^34^. When fused to a target protein, the ODC degron, derived from the rapidly turning over ODC is sufficient to confer proteasome-mediated degradation^48,49^. Importantly, proteasome-mediated ODC degradation is ubiquitin-independent and thus ODC represents a relevant model to help delineate the contribution of the proteasome in tackling un-ubiquitylated TRIB1. Stably transduced HepG2 cells, were generated with either pJA291 (GFP only) or pJA317 (GFP-ODC1), which also express a puromycin-N-Acetyltransferase-mCherry fusion as a stable internal control. Akin to TRIB1, GFP-ODC1, but not native GFP, was readily upregulated by BTZ and was sensitive to CHX, consistent with the presence of residual proteasome activity (Fig. 7A). Furthermore, TRIB1 was more sensitive to CHX. Quantification revealed 50% (± 16%; n = 4) of signal retention for GFP-ODC1 signal versus 24% (± 5%; n = 5) for recombinant TRIB1 (*p* = 0.014, unpaired *t* test). Like TRIB1, higher PI concentrations and various PI combinations failed to further prevent GFP-ODC1 loss to CHX treatment (Fig. 7B). These experiments demonstrated *1-* enduring proteasome activity despite the presence of high inhibitor concentration and *2-* greater instability of TRIB1, which may reflect preferential proteasome mediated degradation or the contribution of proteasome independent degradation pathways.

### TRIB1 proteolysis in response to BTZ occurs independently of the lysosomal system

We hypothesized that autophagy engagement could contribute to BTZ resistant TRIB1 loss. Indeed, proteasome inhibition was previously shown to activate lysosomal-autophagy^23^. A possible contribution of the lysosome was tested using CLQ, an inhibitor of lysosome acidification, which is required to activate resident proteases. Prolonged incubation of HepG2-T1 with CLQ, which increased LC3BII, an indicator of lysosome-autophagy inhibition, did not however prevent recombinant TRIB1 loss, suggesting that TRIB1 loss is independent of autophagy (Figure S10).

### Cytosolically retained TRIB1 is unstable

**Figure 8:**
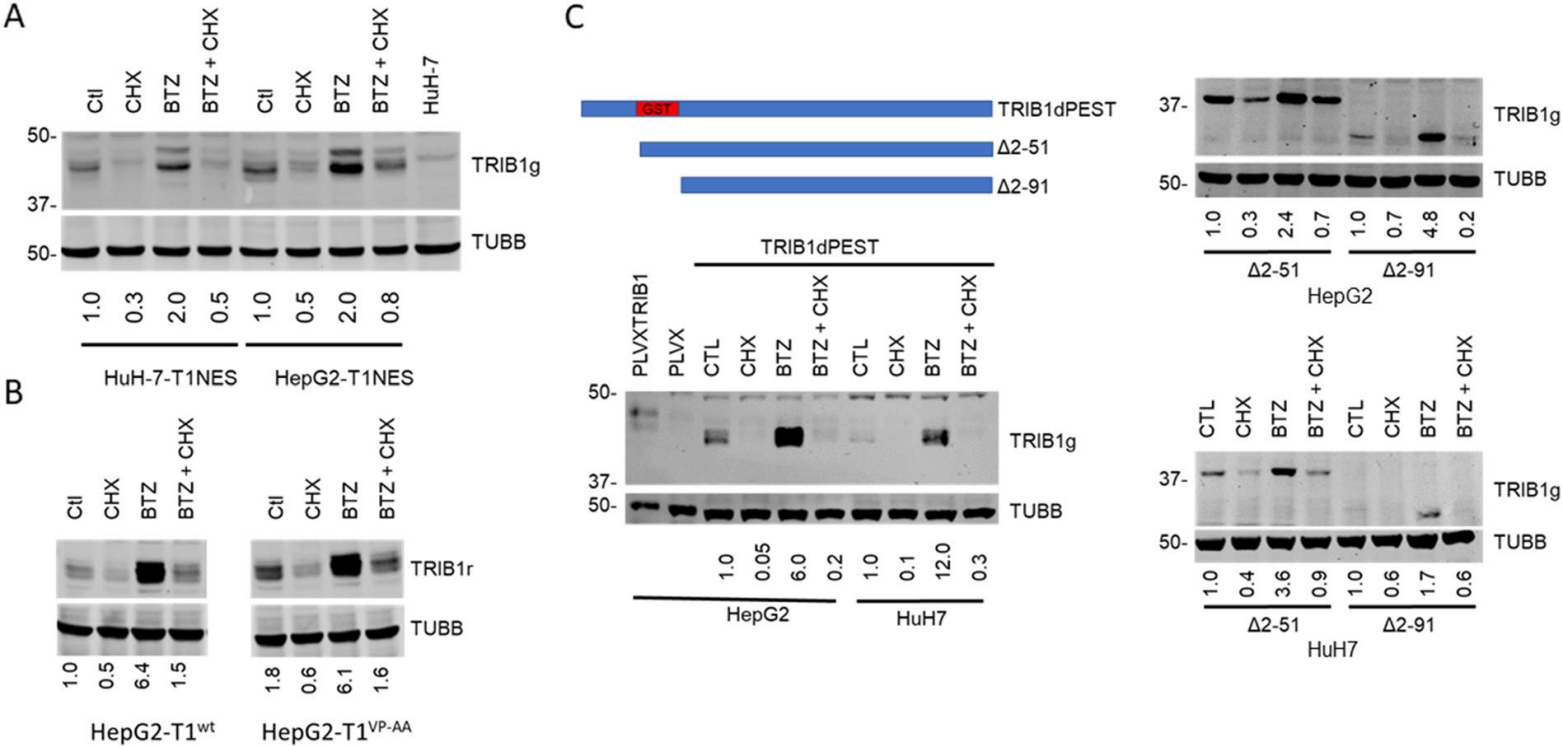
Western blot analyses of TRIB1 mutants and deletions in response to BTZ and CHX. Stable pools of TRIB1 overexpressing HepG2 and HuH-7 cells were generated by lentiviral mediated transduction with PLVXTRIB1NES (**A**), PLVXTRIB1wt or VP-AA (**B**), TRIB1dPEST and N-terminal deletions (**C**). Schematics of the substitution and deletions is depicted. Cells were treated for 5 h with BTZ and/or CHX, as indicated. Data are representative of 2–3 independent experiments. Cell lysates were analyzed by Western blotting using TRIB1 (rabbit(r) or goat (g)) and TUBB antibodies. Quantifications are internally controlled to the matching TUBB signal and are expressed relative to the corresponding Ctl value (1% DMSO) except for (**B**), where values are expressed relative to the T1wt, Ctl value.

Structural features driving TRIB1 instability were next investigated. Instability could result from regulatory events taking place early during TRIB1 synthesis either in the cytosol, after its nuclear import, or both. To test the relative contribution of cytosolic and nuclear processes, we took advantage of a TRIB1 construct wherein the bipartite nuclear localization sequence was replaced by PKIA nuclear export signal. We previously showed using transient transfection that TRIB1 was unstable in HeLa cells. To mitigate issues noted previously related to the greater stability of transiently transfected *TRIB1*, *TRIB1* was transduced by lentiviral delivery. As previously described in non-hepatic cell lines, the protein localized exclusively to the cytoplasm (Figure S11). The inclusion of BTZ resulted in its upregulation and, importantly, TRIB1 was rapidly lost when de novo protein synthesis was blocked with CHX (Fig. 8A). Thus, domains situated beyond the NLS region appear sufficient to render TRIB1 unstable, via cytosol mediated processes.

### COP1 does not regulate TRIB1 protein abundance and turnover

COP1 is an E3 ligase functionally integrated with TRIB1. It normally resides in both nuclear and cytoplasmic compartments and can shuttle between the 2 compartments^50^. To test whether COP1 plays a role in the loss of TRIB1 in the presence of CHX (under conditions where the proteasome is functional), a TRIB1 construct deficient in COP1 binding, as well as a Wild-type version for direct comparison, were transduced in HepG2^51^. Both variants were similarly sensitive to CHX, demonstrating TRIB1 instability does not require its interaction with COP1 (Fig. 8B).

### Instability of TRIB1 does not require the Pro-Glu-Ser-Thr (PEST) region or the N-terminal region of TRIB1

The N-terminal region of TRIB2 was implicated in mediating its instability^52^. TRIB1 contains a predicted disordered N-terminus (https://mobidb.bio.unipd.it/Q96RU8; Alphafold) that is enriched in P, E, S and T residues, typical of PEST domains associated with protein instability^53^. To examine the contribution of the PEST-like region in destabilizing TRIB1, we substituted the domain for 2 amphipathic helices of equivalent lengths derived from the more stable GST protein (Fig. 8C). The substituted TRIB1 was potently upregulated by BTZ but remained highly unstable to CHX, implying that the PEST region is not necessary to confer TRIB1 instability. Similar conclusions were drawn from N-terminal truncations: deletion of either AA 2–51 or AA 2–91 of TRIB1 did not render TRIB1 resistant to CHX (Fig. 8C). Interestingly, the latter deletion constructs did not show the cluster of bands typical of full-length TRIB1, hinting that this feature is dictated by its N-terminal region and is unrelated to its instability.

## Discussion

Proteasome inhibitors are therapeutically important for some cancers. However, as proteasome function is essential to normal cell function, proteasome inhibition is stressful and may be hepatotoxic^17^. By contrast, limited proteasome inhibition can be protective by promoting anti-oxidative pathways^20^. Here we examined the impact of proteasome inhibition on TRIB1, an important regulator of liver function. Proteasome inhibition was accompanied by increased TRIB1 transcription, as well as protein instability under proteasome permissive and non-permissive conditions in hepatocyte models. TRIB1 protein abundance was accompanied by potent transcription upregulation. This differs from our previous work in non-liver models (HeLa, HEK293T and aortic smooth muscle cells) wherein we reported that BTZ increased recombinant TRIB1 protein abundance 2–fourfold without an accompanying increase in *TRIB1* mRNA abundance^33^. The relative contributions of direct (reduced proteolysis) and indirect (increased transcript abundance) TRIB1 regulators in liver models in response to PI is unclear. However, as recombinant TRIB1 transduction was sufficient to increase TRIB1 protein abundance, increased transcript concentration likely played a major role. This is further supported by ACTD treatments, which prevented PI induced TRIB1 protein increases.

More studies will be needed to identify the effectors involved in TRIB1 transcript upregulation. ER stress response genes are likely involved. BTZ induced ER stress, as reflected by increased CHOP expression, a major stress response transcription factor previously linked to TRIB3 upregulation^41^. However, unlike TRIB3, TRIB1 was only mildly impacted by Tunicamycin, a potent ER stressor, suggesting that ER stress plays an accessory role. We showed that *ATF3* was needed for basal *TRIB1* expression and for maximal *TRIB1* response to PIs. Whether PI treatment led to increased ATF3 occupancy at the TRIB1 promoter in response to PIs was not examined*.* Previous studies demonstrated that liver-specific *Trib1* deficiency led to increased, CEBPA dependent, *ATF3* expression^5^. Our work complements these findings by demonstrating that *ATF3* promotes *TRIB1* expression. Thus, by contributing to the maintenance of *TRIB1* levels and thus lower CEBPA abundance, ATF3 enforces a negative feedback loop on its own expression.

The proteasome is responsible for the vast majority of protein degradation, including most short lived proteins^54^. Co-translational degradation via the proteasome is widespread and contributes to protein synthesis quality control^55–57^. It has been estimated that 12–15% of nascent polypeptides are ubiquitylated in HEK293T^55^. De novo co-translated proteins are ubiquitylated and can be readily stabilized by proteasome inhibition. Similarly, we observed that polyUb signals, resulting from the incorporation and accumulation of Ub chains in cellular proteins, were increased and stabilized by PI. By contrast, TRIB1 was only partially stabilized by proteasome inhibitors, as revealed by delayed but still rapid TRIB1 loss. Loss could be due to residual proteasome activity, as suggested by the lingering instability of the GFP-ODC1 reporter in the presence of PI. However, the contribution of non-proteasome pathways cannot be excluded. Unfortunately, inability to fully block proteasome activity impedes the investigation of these putative alternate degradation routes.

Rapid proteasome-mediated loss of TRIB1 (and GFP-ODC1) raises the prospect of a preferential earmarking of TRIB1 for degradation vis-à-vis the bulk of proteasome targets, perhaps related to its lack of ubiquitylation. Preferential degradation of a non-ubiquitylated vs ubiquitylated target has been reported: whereas heat shock failed to stabilize Un-ubiquitylated GFP-ODC1, a ubiquitylated form of GFP was fully stabilized^58^.

In contrast to TRIB1, TRIB2, which has been linked to liver tumorigenesis, is ubiquitylated via smuf1 and/or βTRCP^52,59,60^. Interestingly, TRIB2 instability to CHX treatment was largely abrogated by removal of its N-terminal 50 AA, whereas TRIB1 deletions spanning the N-terminus as well as substitutions of AA 31 to 88 (NLS + PEST regions) were insufficient to stabilize it. In addition, TRIB2 can be destabilized pharmacologically via an interaction between its pseudo kinase domain and a family of EGFR inhibitors^61^. As all tribbles share structurally well conserved pseudo kinase domains, this domain may render TRIB1 unstable as well.

TRIB1 instability was independent of its interaction with COP1, as suggested by the propensity of the binding deficient variant to remain unstable. Rapid TRIB1 turnover might stem from quality control events, perhaps to address a propensity of TRIB1 to misfold. Indeed, our results indicate that TRIB1 loss occurs in the cytosol, which suggests that degradation precedes its import in the nucleus, perhaps alongside COP1^50^. Whether all losses occur in the cytosol is unknown. TRIB1 may be specifically turned over via regulatory feed-back loops to ensure a timely adaptation to changing cellular conditions. Given the role of TRIB1 in cell growth and proliferation, one could envision interactors either stabilizing or destabilizing the emerging TRIB1 to match nutrient abundance. Instability could be mediated via post-translational modifications (PTMs). For instance, phosphorylation of cyclin D1 and AKT, impairs their degradation^57,62^. Relatively little is known about TRIB1 PTMs. TRIB1 migration on SDS-PAGE, characterized by several closely migrating bands, is certainly consistent with low molecular weight covalent modifications. In preliminary experiments, phosphatase treatment of whole cell lysate failed to impact this pattern, suggesting that it does not arise from alternative phosphorylation events. Interestingly, an analogous pattern was observed with the terminal PEST- and NLS-substituted TRIB1 constructs (AA 53–88 and AA 30–51, respectively) but not with the Δ2-51 N-terminal deletion, implying that this pattern requires AA 2–29 of TRIB1.

## Supplementary Information

Supplementary Information 1.

Supplementary Information 2.

## Data Availability

The datasets analyzed by Enrichr during the current study are available in the Gene Expression Omnibus (GEO), GSE166923.
