# Supplementary material for: Regulation of TRIB1 abundance in hepatocyte models in response to proteasome inhibition
Source: Sci Rep. 2023 Jun 8;13:9320. doi: 10.1038/s41598-023-36512-7 (PMC10250549; doi:10.1038/s41598-023-36512-7)

Original gels: listed by figure numbers, with highlighted regions corresponding to figures in manuscript

Sequential probing shown in greyscale and coincubations shown with false colors.

Fig 1 A

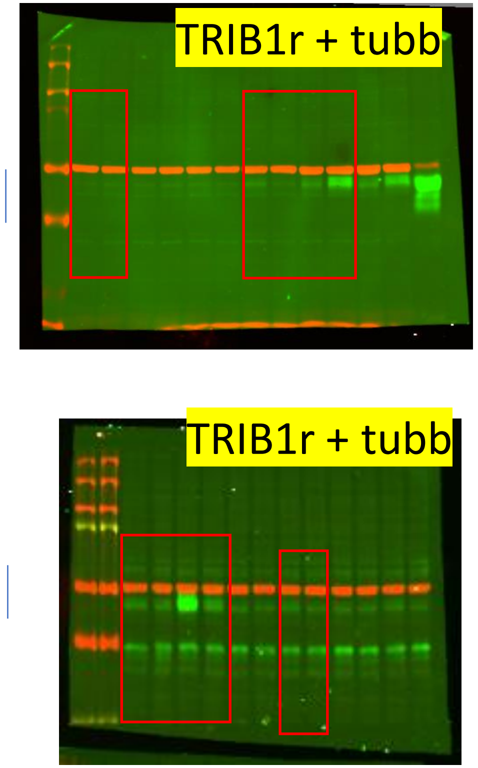

Fig 1 B

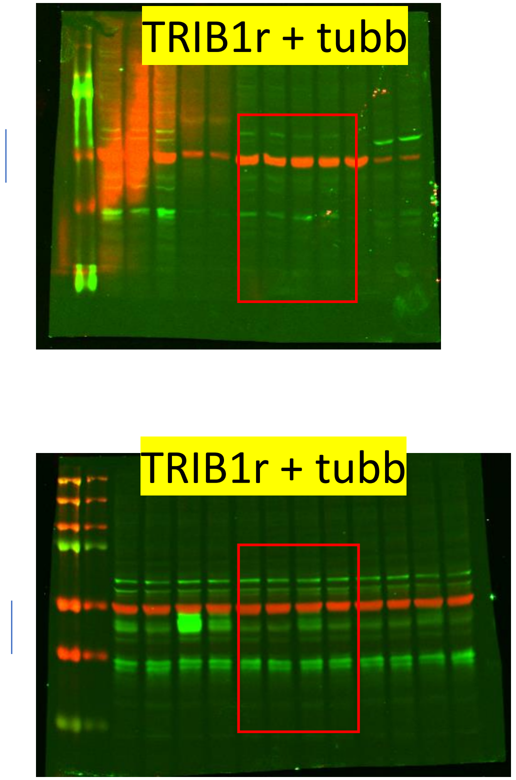

Fig 1 C

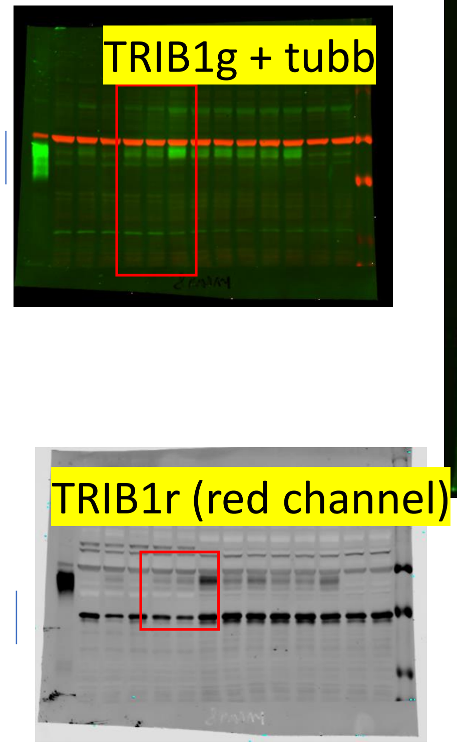

Fig 1 D

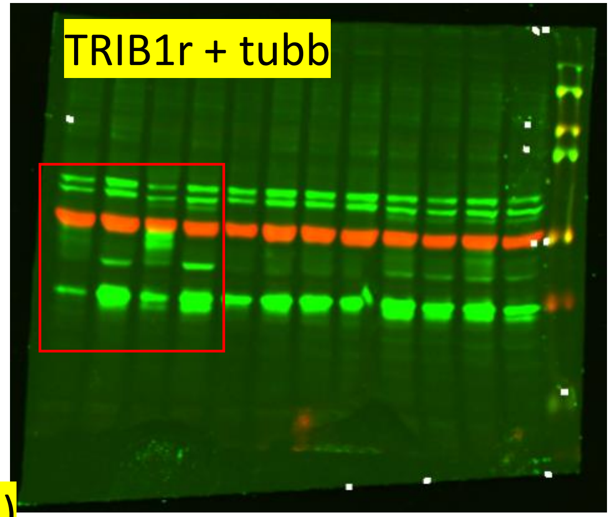

Fig 2B

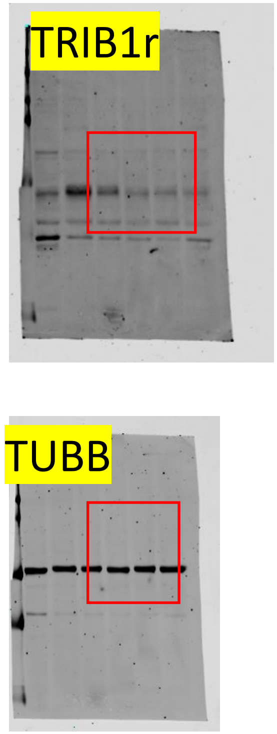

4A

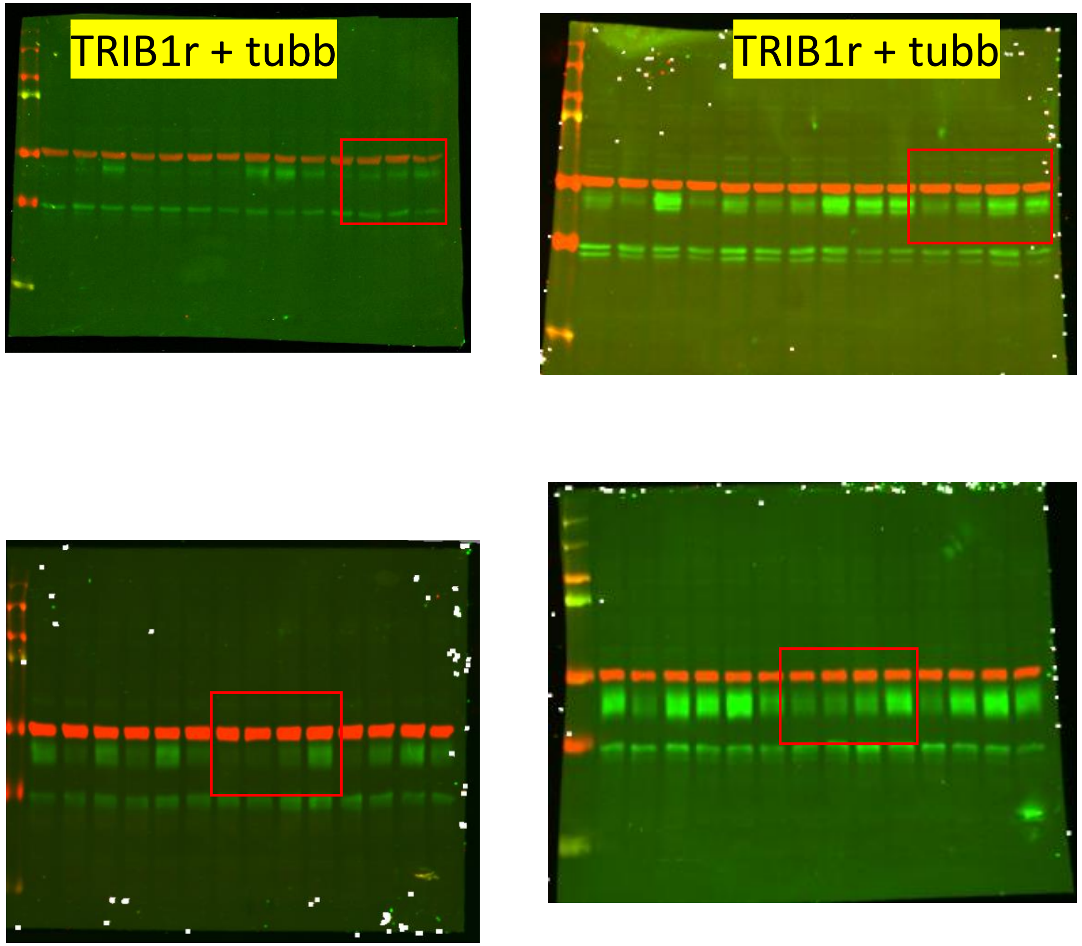

4B

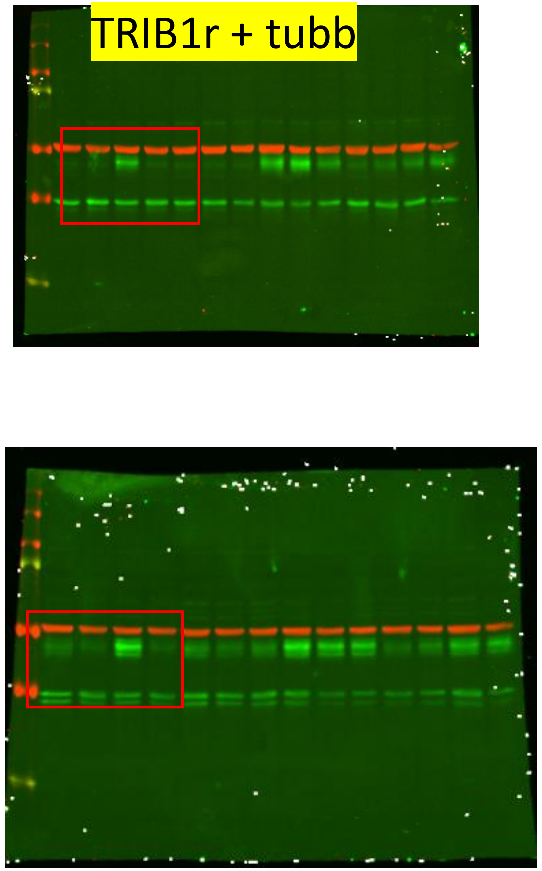

Fig 5

A

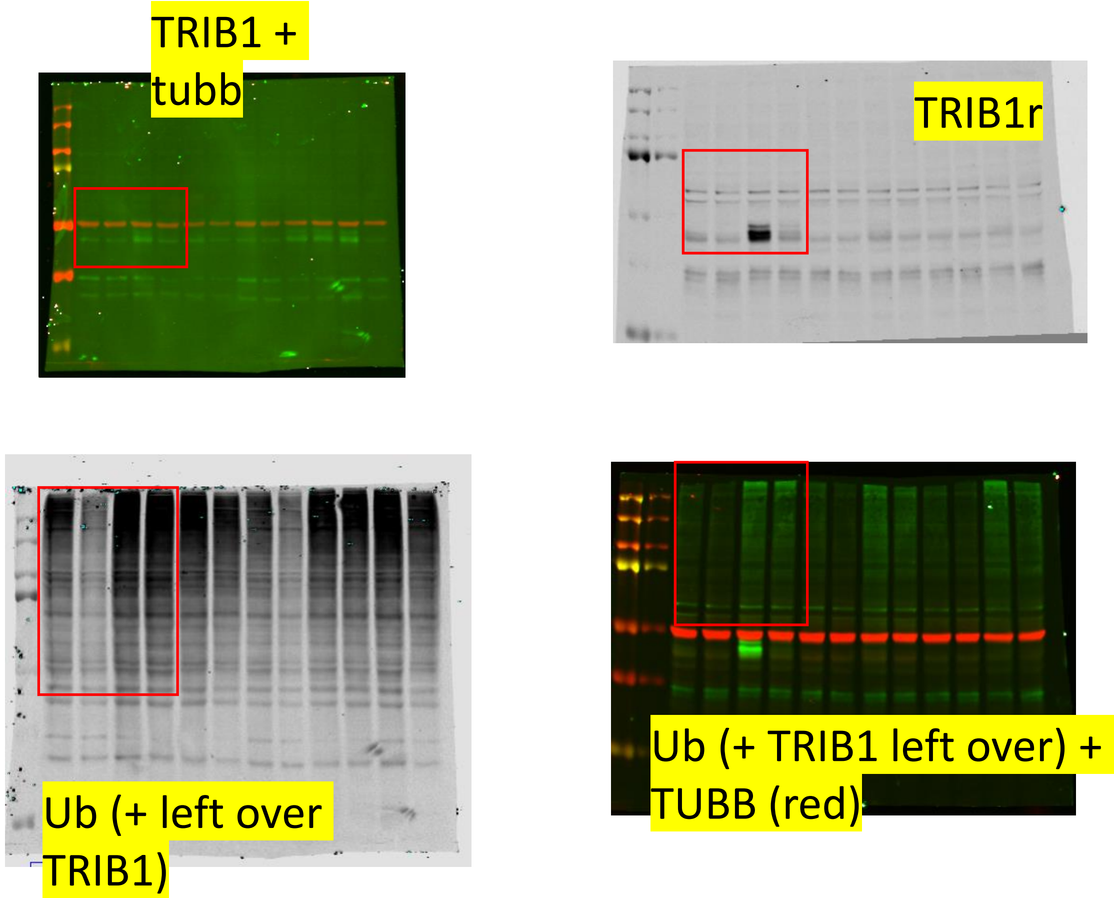
TRIB1 + tubb
TRIB1r
Ub (+ left over TRIB1)
Ub (+ TRIB1 left over) + TUBB (red)

Fig 6

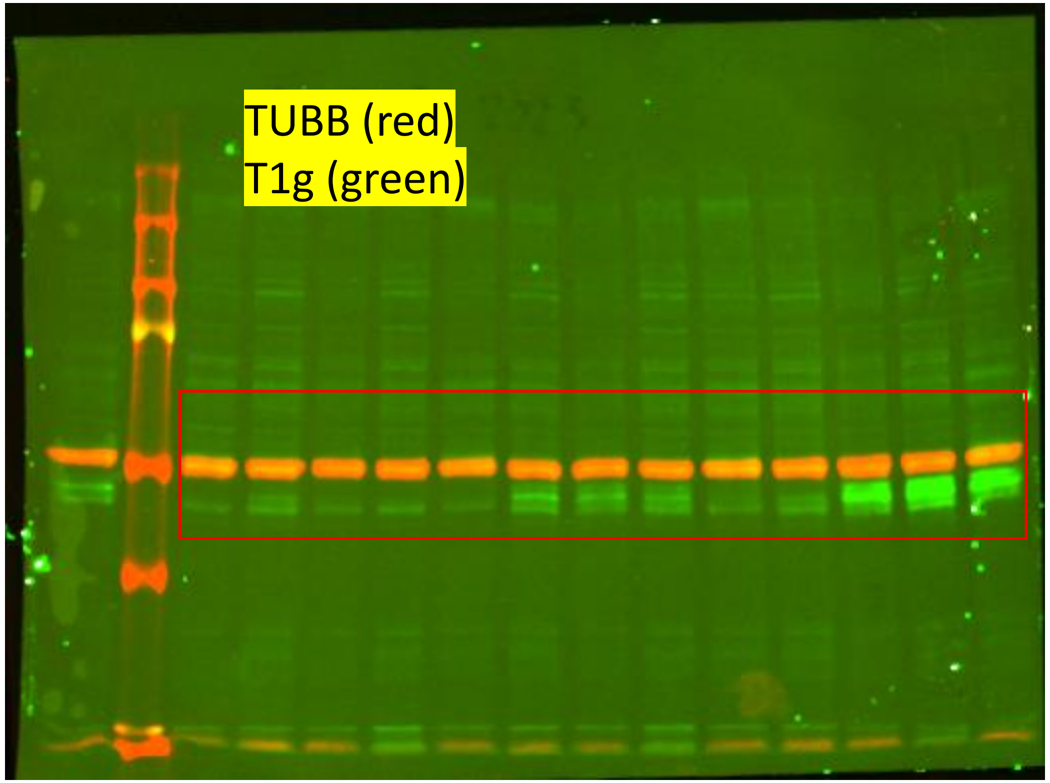

Fig 7A

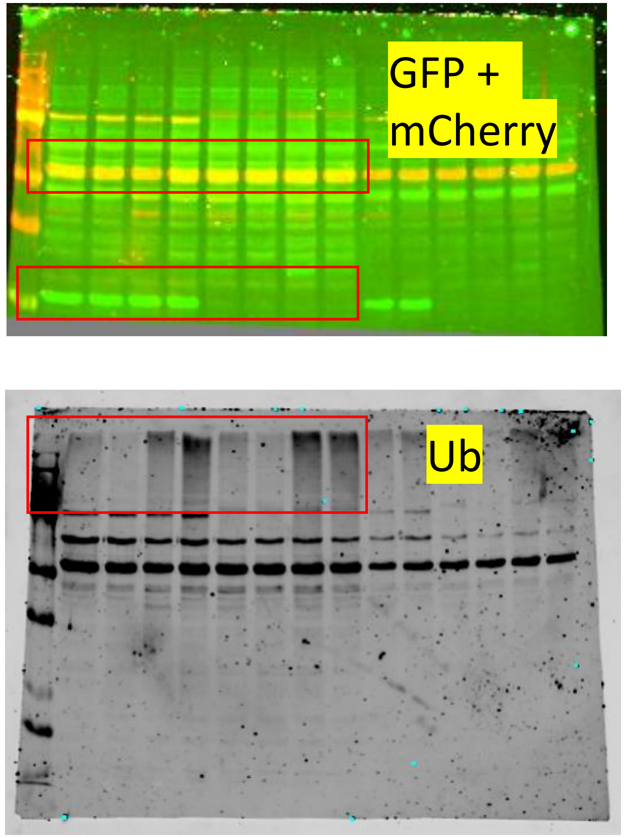

Fig 7B

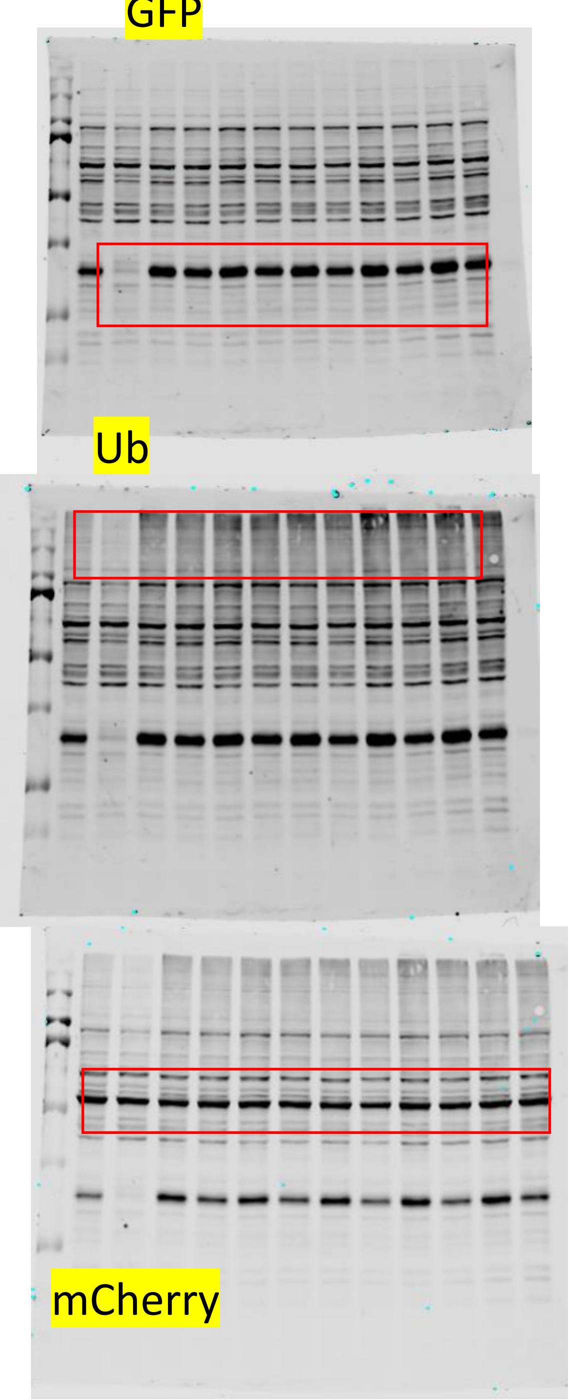

Fig 8A

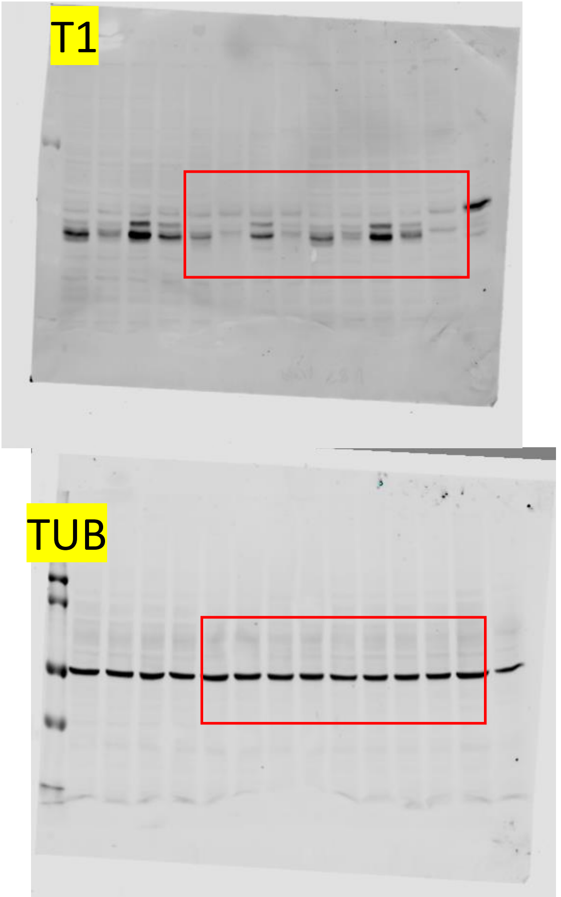

Fig 8B

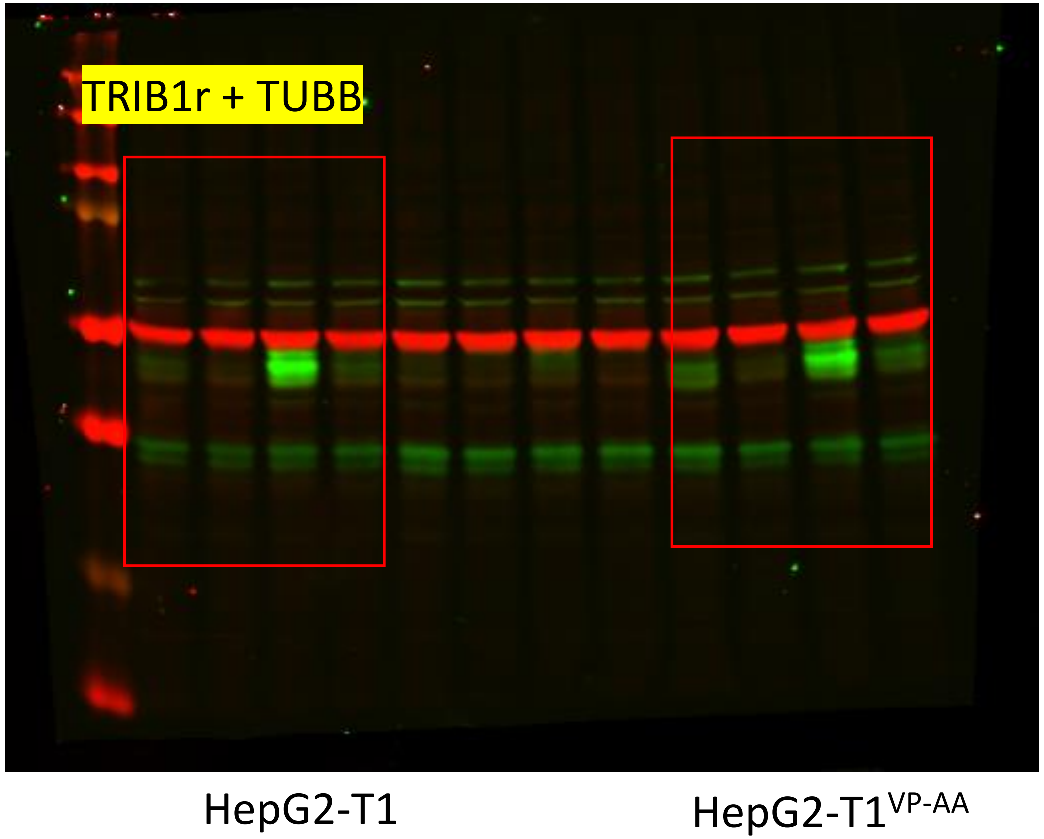

Fig 8C

TRIB1g (red)

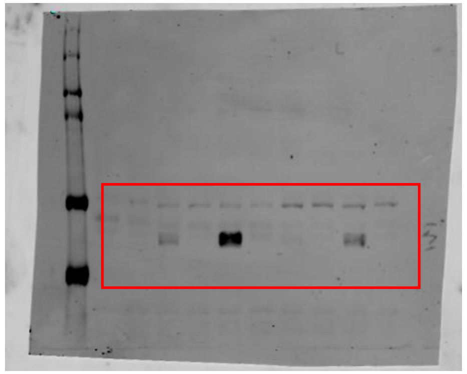

TUBB

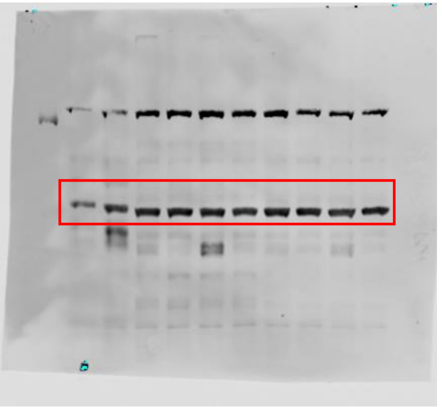

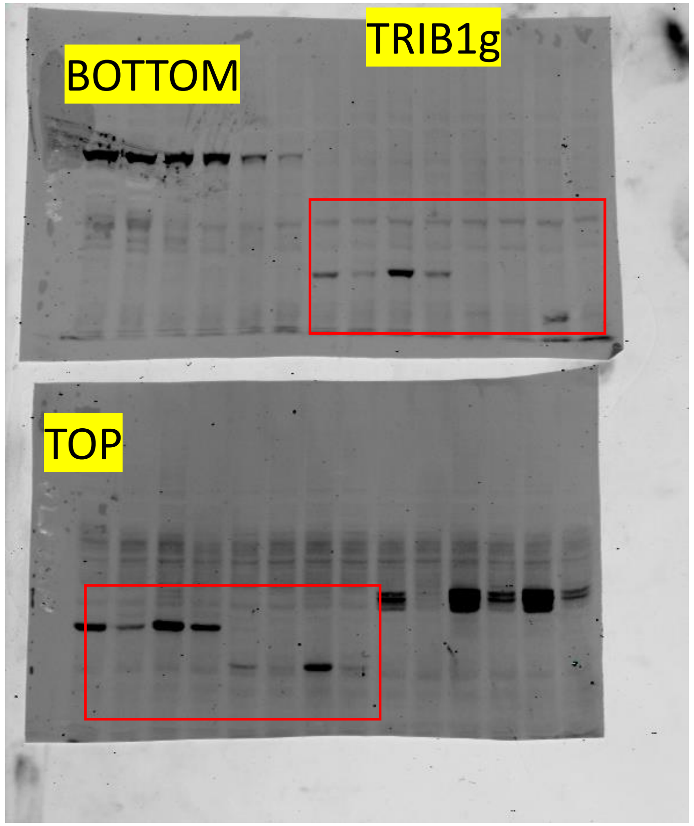

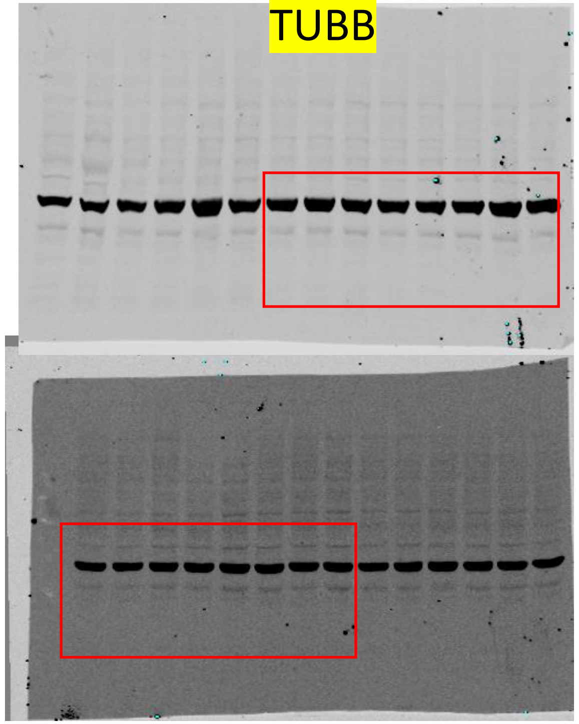

Fig S2

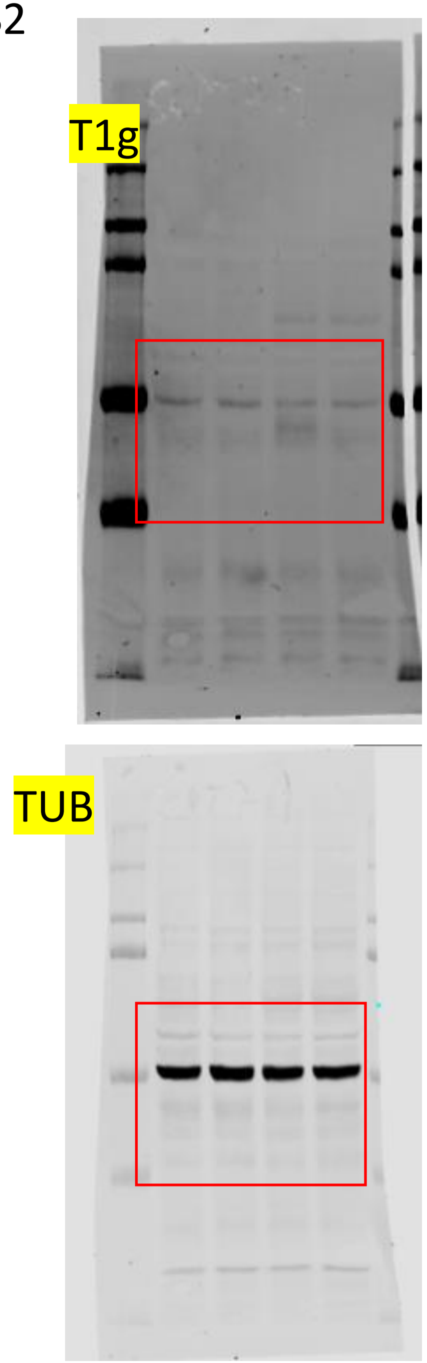

Fig S6

A

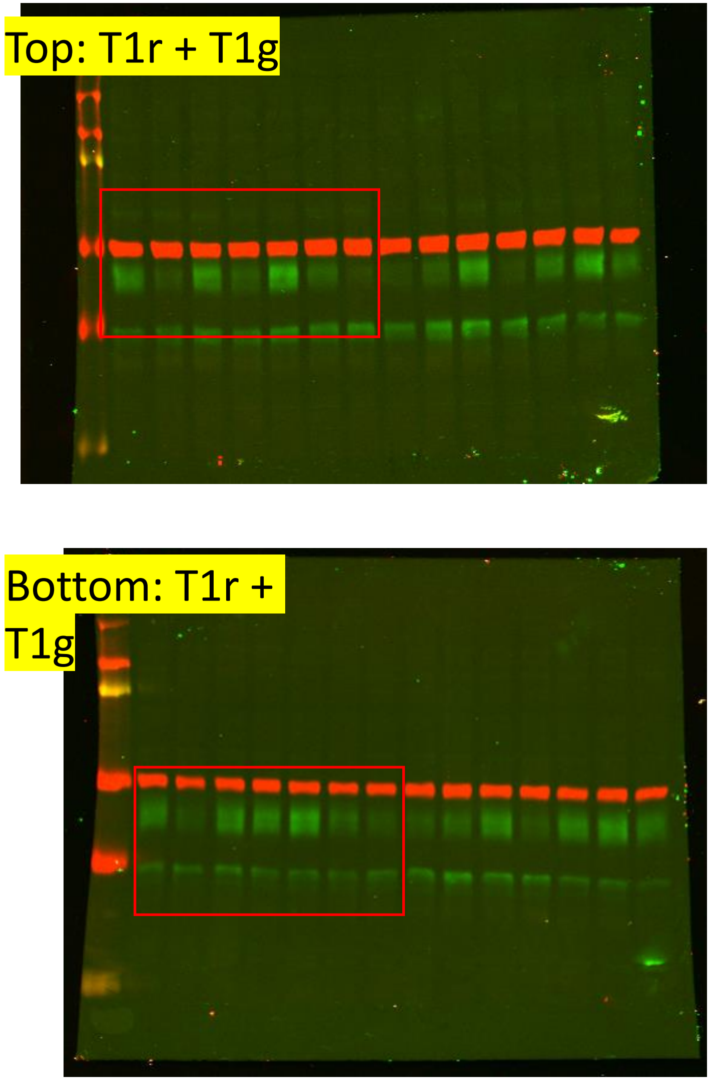

Fig S7

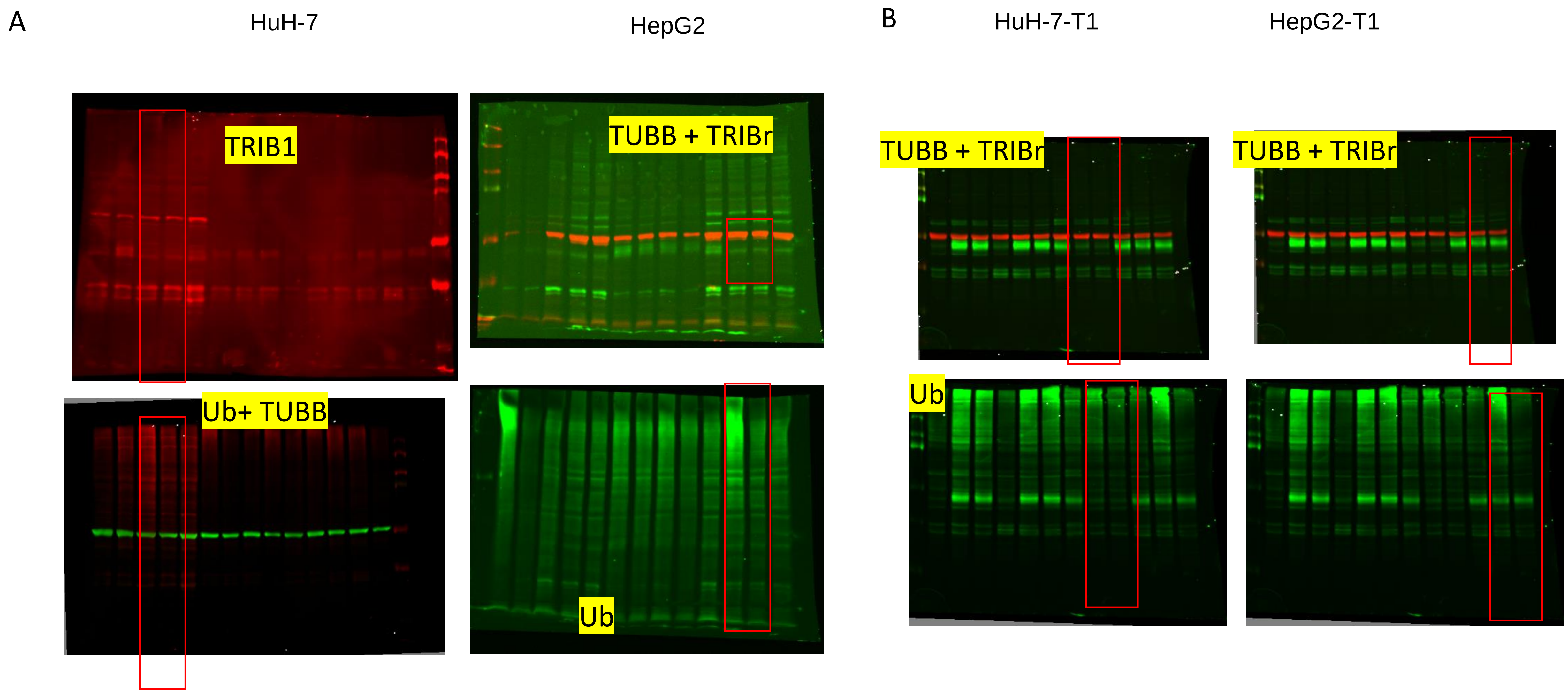
A
HuH-7
HepG2
TRIB1
TUBB + TRIBr
Ub+ TUBB
Ub
B
HuH-7-T1
HepG2-T1
TUBB + TRIBr
TUBB + TRIBr
Ub

Fig S8

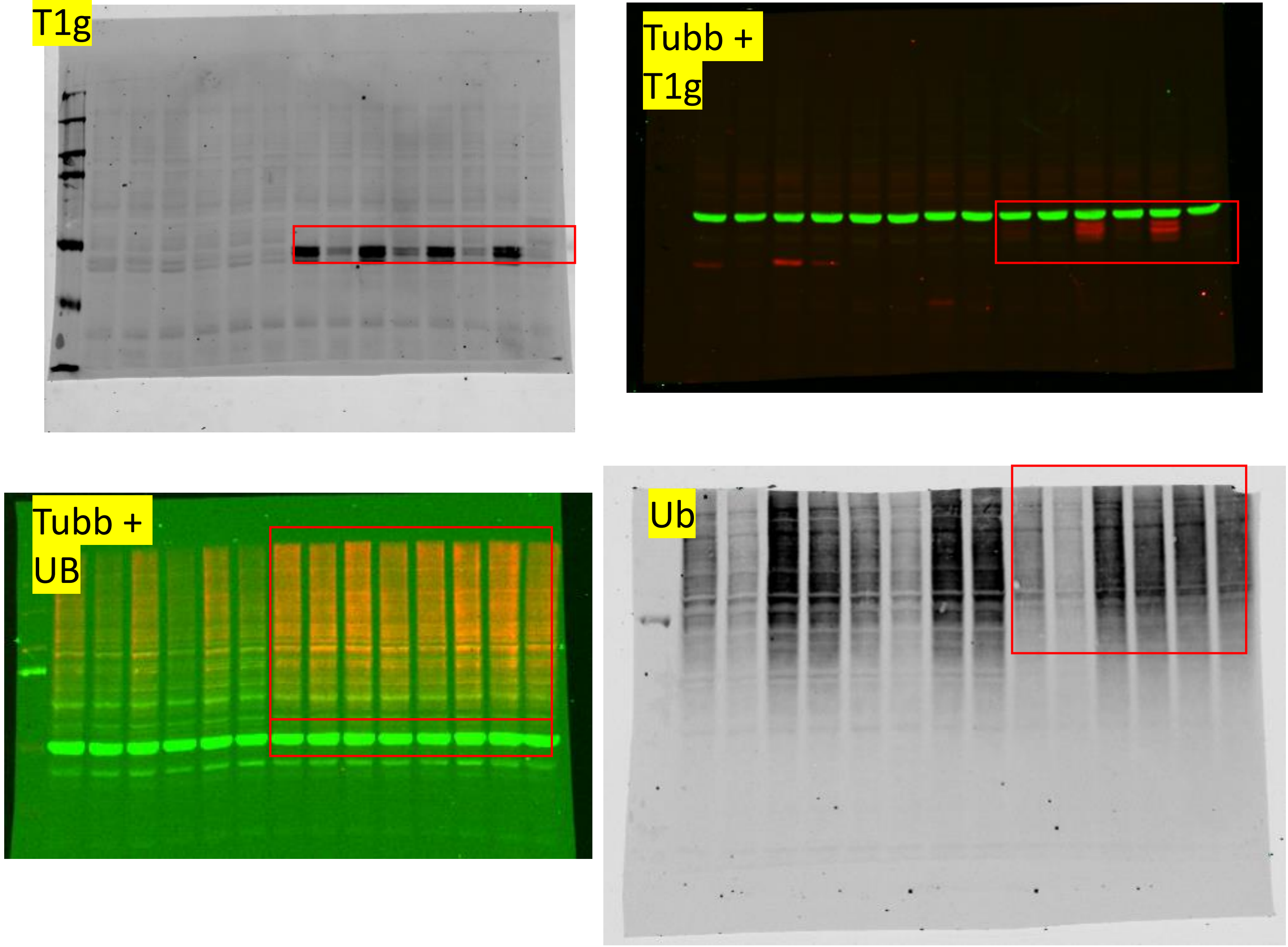

Fig S9

A

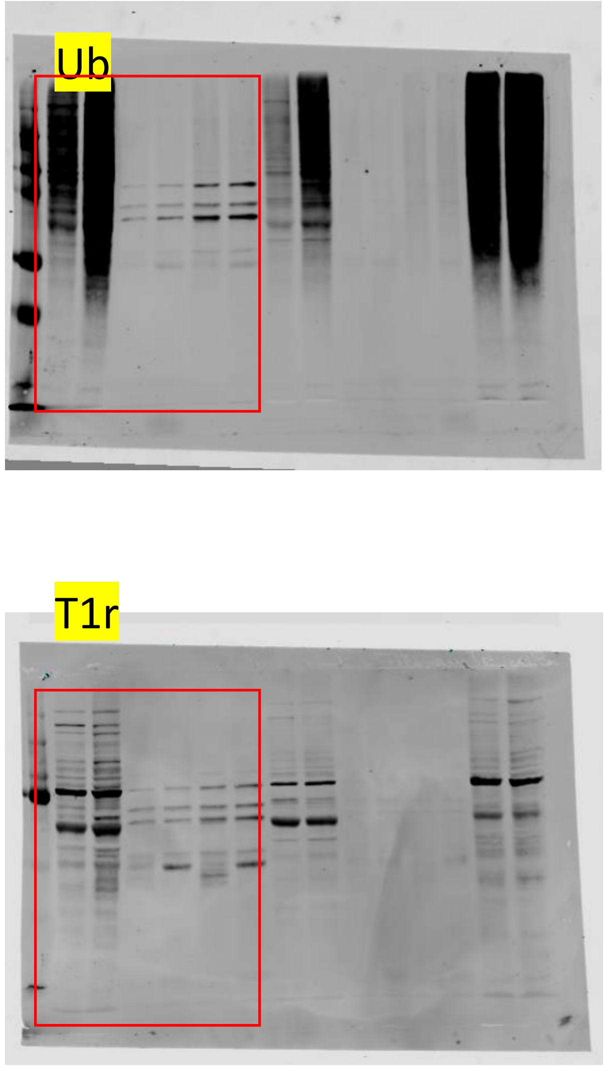

B

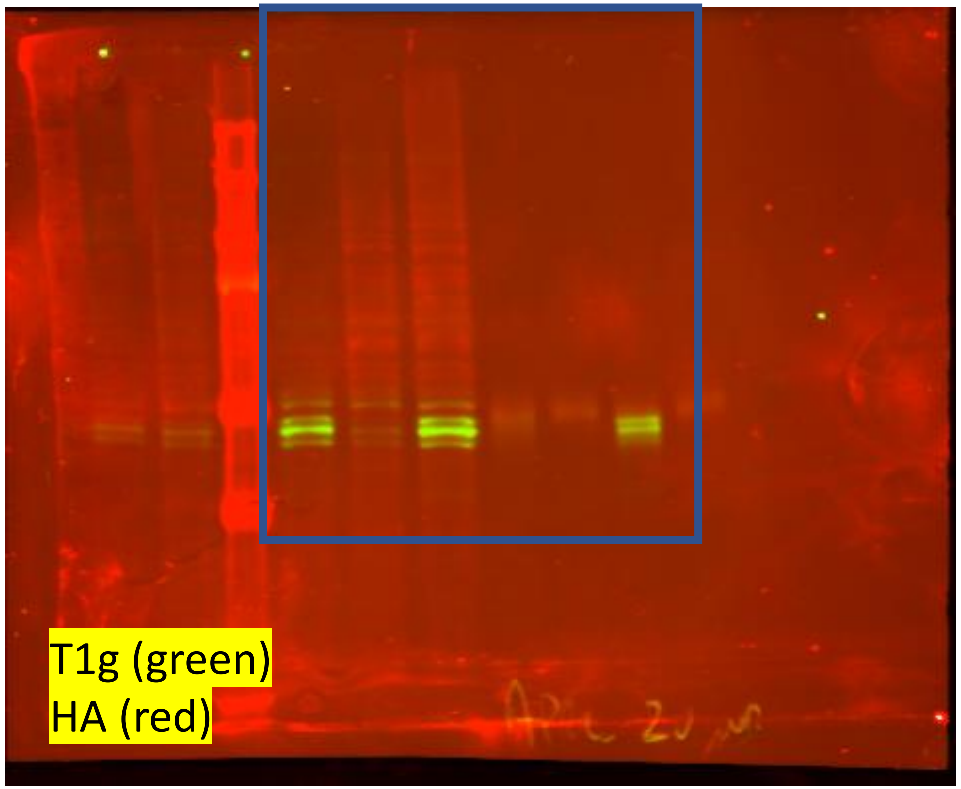

Fig S10

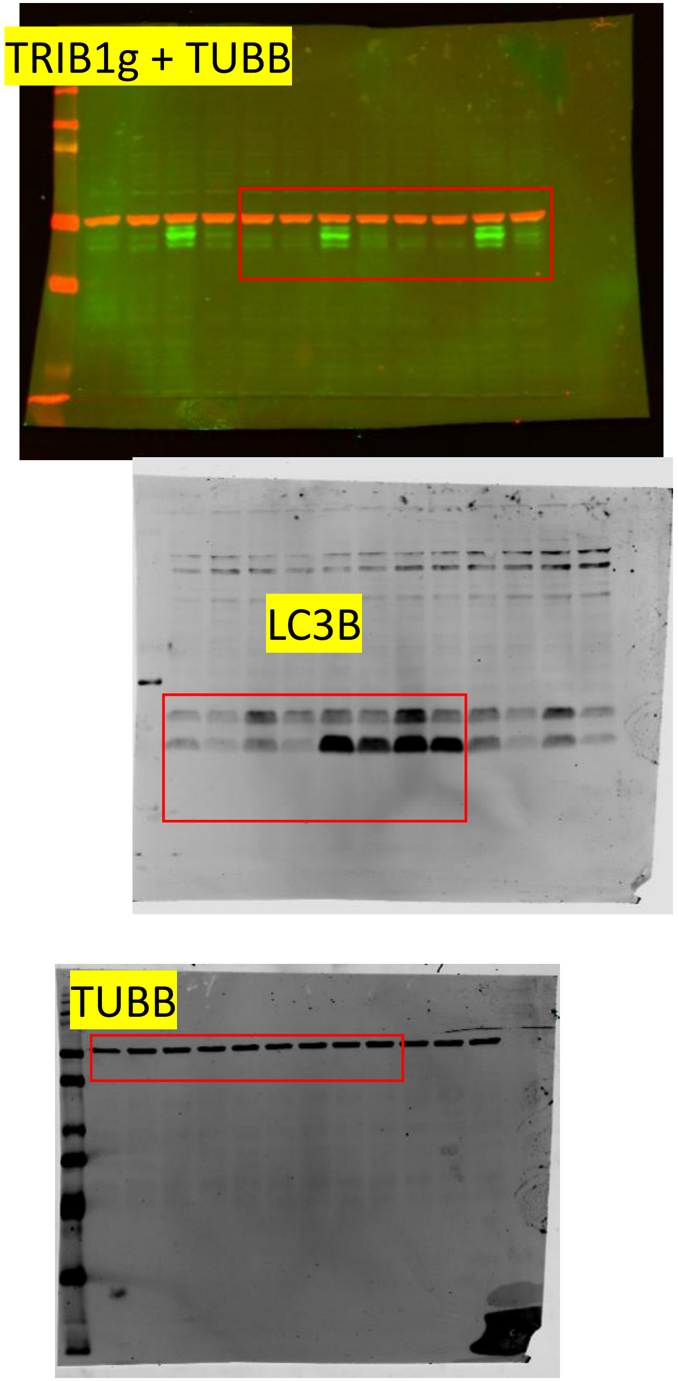

Supplement: Supplementary file 1 — Supplementary Information 1. [file 41598_2023_36512_MOESM1_ESM.pdf]
