# Supplementary material for: Regulation of TRIB1 abundance in hepatocyte models in response to proteasome inhibition
Source: Sci Rep. 2023 Jun 8;13:9320. doi: 10.1038/s41598-023-36512-7 (PMC10250549; doi:10.1038/s41598-023-36512-7)
Supplement: Supplementary file 2 — Supplementary Information 2. [file 41598_2023_36512_MOESM2_ESM.pdf]

Regulation of TRIB1 abundance in hepatocyte models in response to proteasome inhibition

Sébastien Soubeyrand, Paulina Lau, and Ruth McPherson.

Supplementary Figures

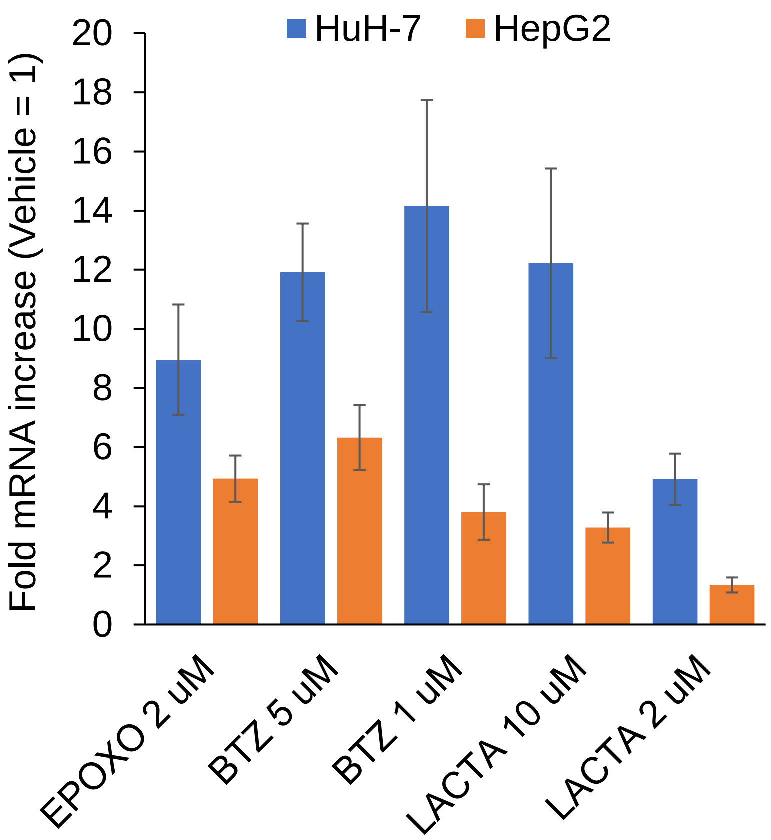

Figure S1. TRIB1 mRNA increases in response to proteasome inhibitors in HuH-7 and HepG2 cells. TRIB1 mRNA abundance (normalized to PPIA level) was measured following 5 h regimen with the indicated drugs (EPOXO, Epoxomicin; BTZ, Bortezomib, LACTA, Lactacystin). Values are expressed relative to the vehicle (1 % DMSO). Bars represent the average of 3 biological replicates (± S.D.) All changes from vehicle (except for LACTA 2 µM; HepG2) were statistically significant (Student's paired t-test, $p<0.05$)

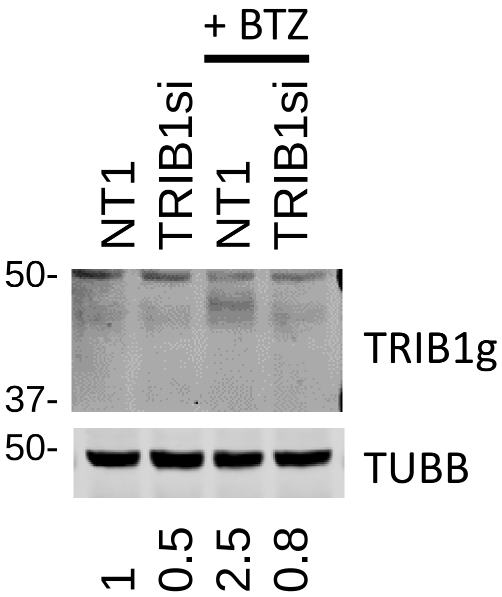

Figure S2. Appearance of the endogenous TRIB1 signal in response BTZ is sensitive to TRIB1 silencing. Western blot analysis of HepG2 cells incubated with a TRIB1 siRNA or a non-target control (NT1) for 72 h and treated with BTZ (5 µM; 16 h). Validation could only be performed for the TRIB1g antibody, as TRIB1r lots available at the time of validation were uninformative due to high background. Data representative of 2 biological replicates.

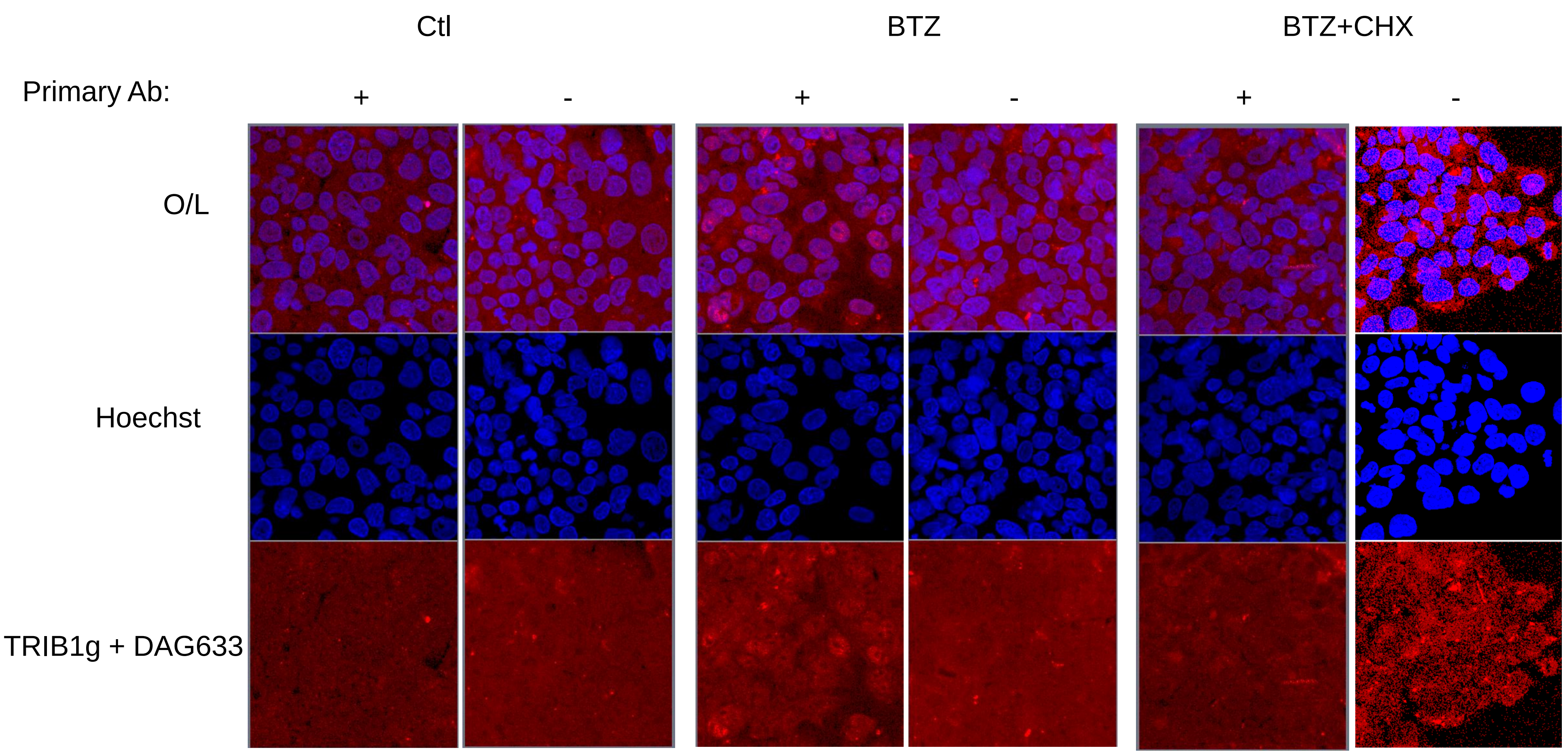

Figure S3. Endogenous TRIB1 signal is specific, nuclear and is detectable in HepG2 cells following a 5 h BTZ treatment. HepG2 cells were seeded on coverslips and treated as indicated with BTZ (5 µM) and/or CHX (10 µg/ml). Cells were then fixed and permeabilized prior to incubations with either a TRIB1 antibody (1:500, +) or no antibody (-) for 1 h. Cells were then washed and incubated for 1 h with a Donkey anti-goat (DAG633) secondary Ab (1:2000) and counterstained with Hoechst. Images were smoothed with a Gaussian filter. Experiment was performed three times with similar results.

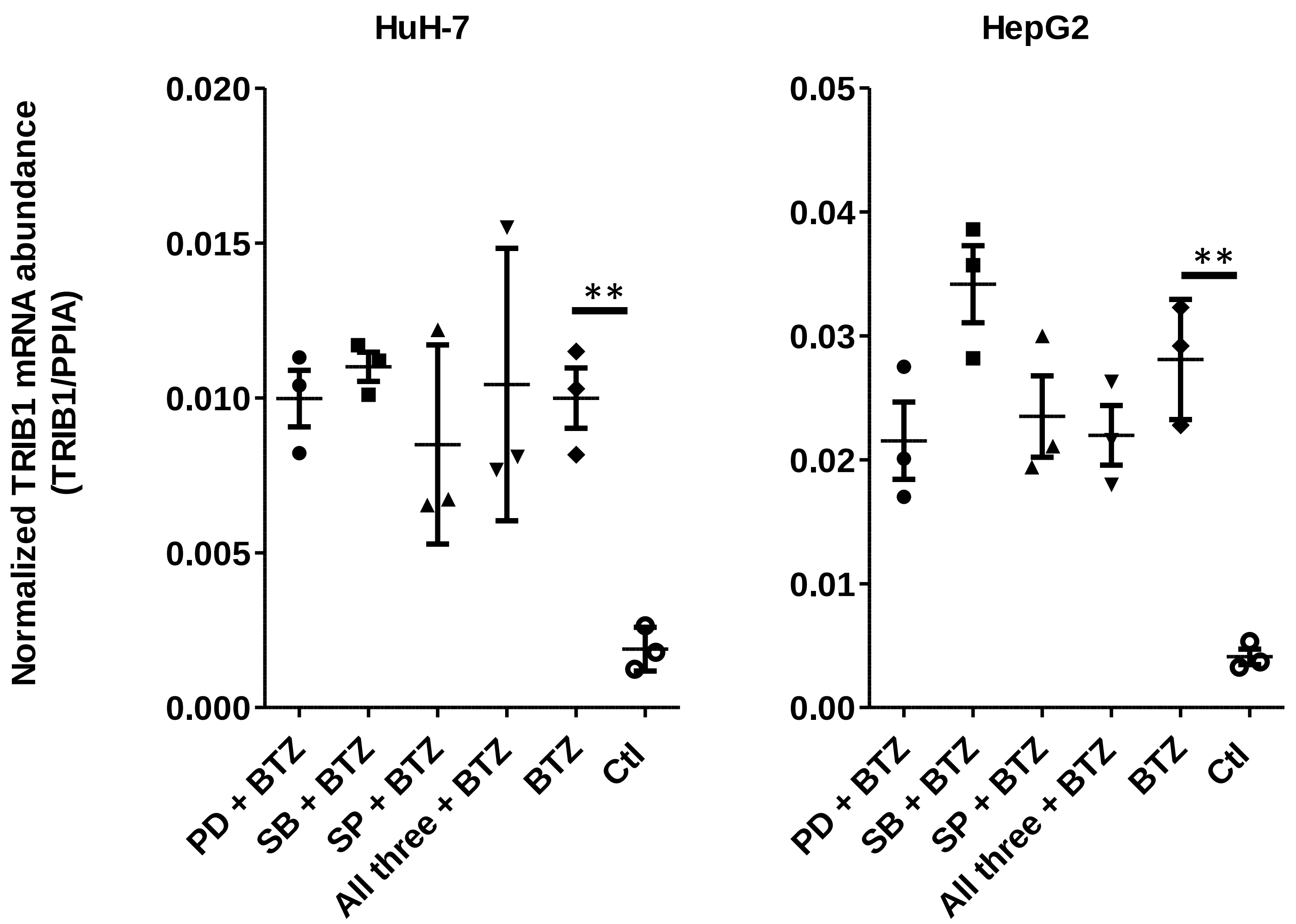

Figure S4. BTZ mediated increased mRNA abundance is resistant to MAPK inhibitors. HuH-7 and HepG2 cells were treated with BTZ for 5 h in the presence of MAPK inhibitors (PD: PD98059 (ERK1/2), SB: SB203580 (P38), SP: SP600125 (JNK)), either singly or in combination, as indicated; Ctl cells were treated with vehicle only (1% DMSO). Experiment was repeated 3 times and individual replicates are shown (± SD). TRIB1 expression is normalized to PPIA levels. Differences between [MAPKi + BTZ] treatments and BTZ were not statistically significant (one-way repeated measures ANOVA with Dunn's post-hoc test vs Ctl).

A

Transcription factor	Combined Score	Adjusted P-value	Fold increase (BTZ/DMSO)
ATF3 27146783 Chip-Seq COLON Human	25.97	3.62E-04	26.53
ATF3 23680149 ChIP-Seq GBM1-GSC Human	17.26	9.39E-03	26.53
NRF2 31884422 ChIP-Seq A549 Human Lung Carcinoma	16.49	9.95E-03	0.64
P300 27058665 Chip-Seq ZR-75-30cells Human	14.54	1.76E-02	0.83
RELB 30642670 ChIP-Seq CTB1 Human Placenta Inflammation	25.78	3.07E-02	1.49
RELA 24523406 ChIP-Seq FIBROSARCOMA Human	13.51	3.07E-02	0.82
JUN 26020271 ChIP-Seq SMOOTH MUSCLE Human	12.52	3.07E-02	3.35
JUND 26020271 ChIP-Seq SMOOTH MUSCLE Human	12.22	3.18E-02	1.30
Nerf2 26677805 Chip-Seq MACROPHAGESS Mouse	12.91	3.44E-02	0.64
SMAD2 18955504 ChIP-ChIP HaCaT Human	11.18	4.16E-02	1.14
SMAD3 18955504 ChIP-ChIP HaCaT Human	11.18	4.16E-02	0.57

B

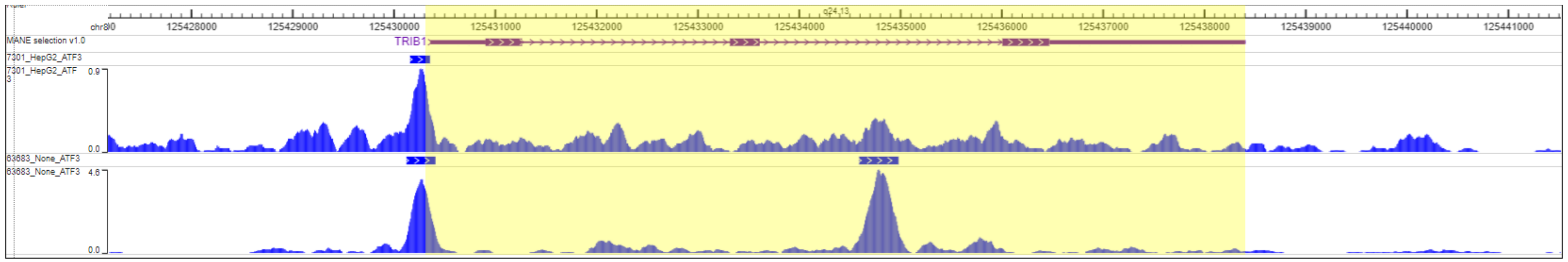

C

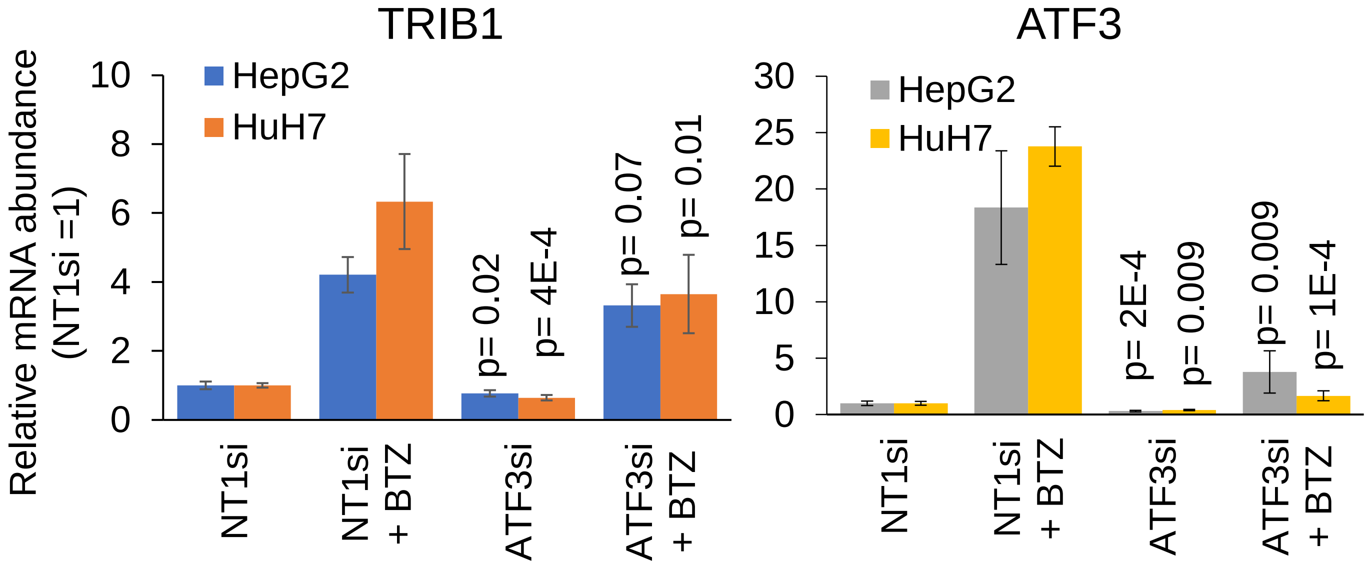

Figure S5. Implication of ATF3 in the regulation of TRIB1 expression. (A) Enrichr analysis of BTZ upregulated genes. Genes that were upregulated (>2-fold) in response to BTZ were analyzed by ChEA through Enrichr (https://maayanlab.cloud/Enrichr/). Only significant hits (Bonferroni adjP<0.05) are shown. Orange highlights indicate transcription factor datasets where *TRIB1* gene was present. Fold-increase in the GSE166923 dataset are shown on the right (nominally significant (Unpaired Student's t-test, p<0.05) changes are in red). (B) WashU Epigenome Browser snapshot of ATF3 binding to the *TRIB1* gene in HepG2 cells. Two sets of data (7301, PMID: 24076218 and 63683, PMID: 29126249) are shown. Data obtained through the Cistrome Data Browser (http://cistrome.org/db/#/). (C) *ATF3* suppression (72 h) reduces TRIB1 expression in HepG2 and HuH-7 cells. Following a 72 h suppression, cells were treated with vehicle or BTZ (5 uM) for 5 h. RNA abundance (left, TRIB1; right, ATF3) was measured by qRT-PCR and corrected for PPIA expression levels. Statistical significance (matching ATF3si vs NT1si treatments) was assessed by unpaired Student's t-tests. P values are shown. Data represent the average of 4 biological replicates (± S.D.).

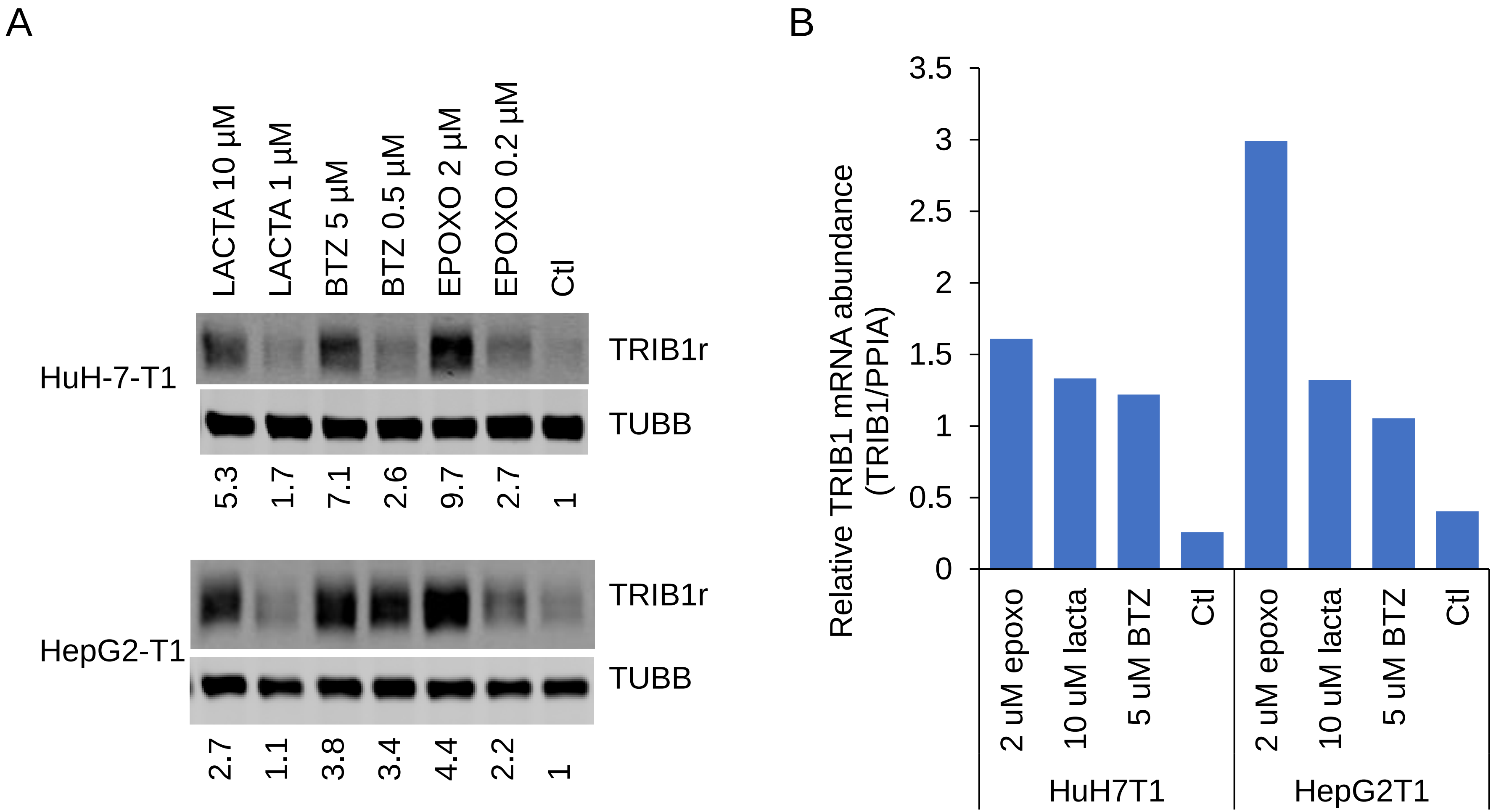

Figure S6. Increased recTRIB1 upon proteasome treatment. HuH-7 and HepG2 cells stably expressing TRIB1 were incubated with vehicle (Ctl), LACTA, BTZ or EPOXO at the indicated concentrations for 5 h. (A) Protein abundance assessed by Western blot. (B) mRNA abundance as measured by qRT-PCR and are expressed relative to PPIA (ΔΔCT method). Data representative of 2 experiments.

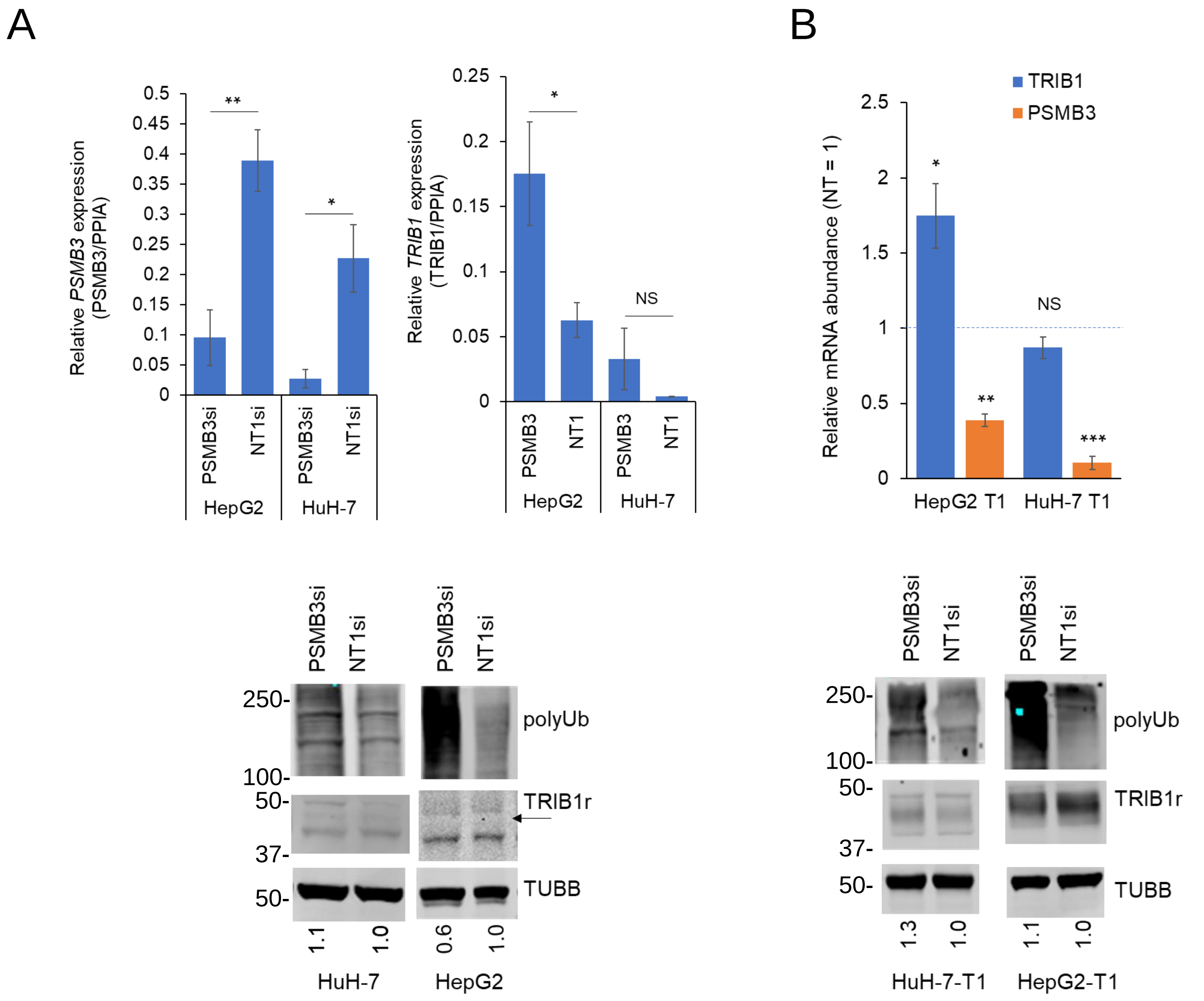

Figure S7. Impact of long-term proteasome suppression on TRIB1 abundance. HepG2 and HuH-7 (A) or their TRIB1 overexpressing stable derivatives (B) were treated for 96 h with a siRNA targeting PSMB3 or a non-target control (NT1). Top, PSMB3 and TRIB1 mRNA abundance was measured by qRT-PCR and bottom, Western blot of corresponding samples. mRNA levels were normalized to PPIA values and are the expressed in raw value (A) or relative to the non-specific control (B). Bars represent the means of 3 biological replicates (± S.D.). Statistical significance for each cell type was tested using a paired Student t-test (NT1si vs PSMB3si). *, P<0.05; **, P<0.01; ***, P< 0.001. NS, difference is not statistically significant. For Western blot analysis of parental cells, arrow points to the predicted position of TRIB1. Quantification represents the TUBB corrected TRIB1 levels, normalized to the NT control value. The experiment was repeated 3 times with similar results.

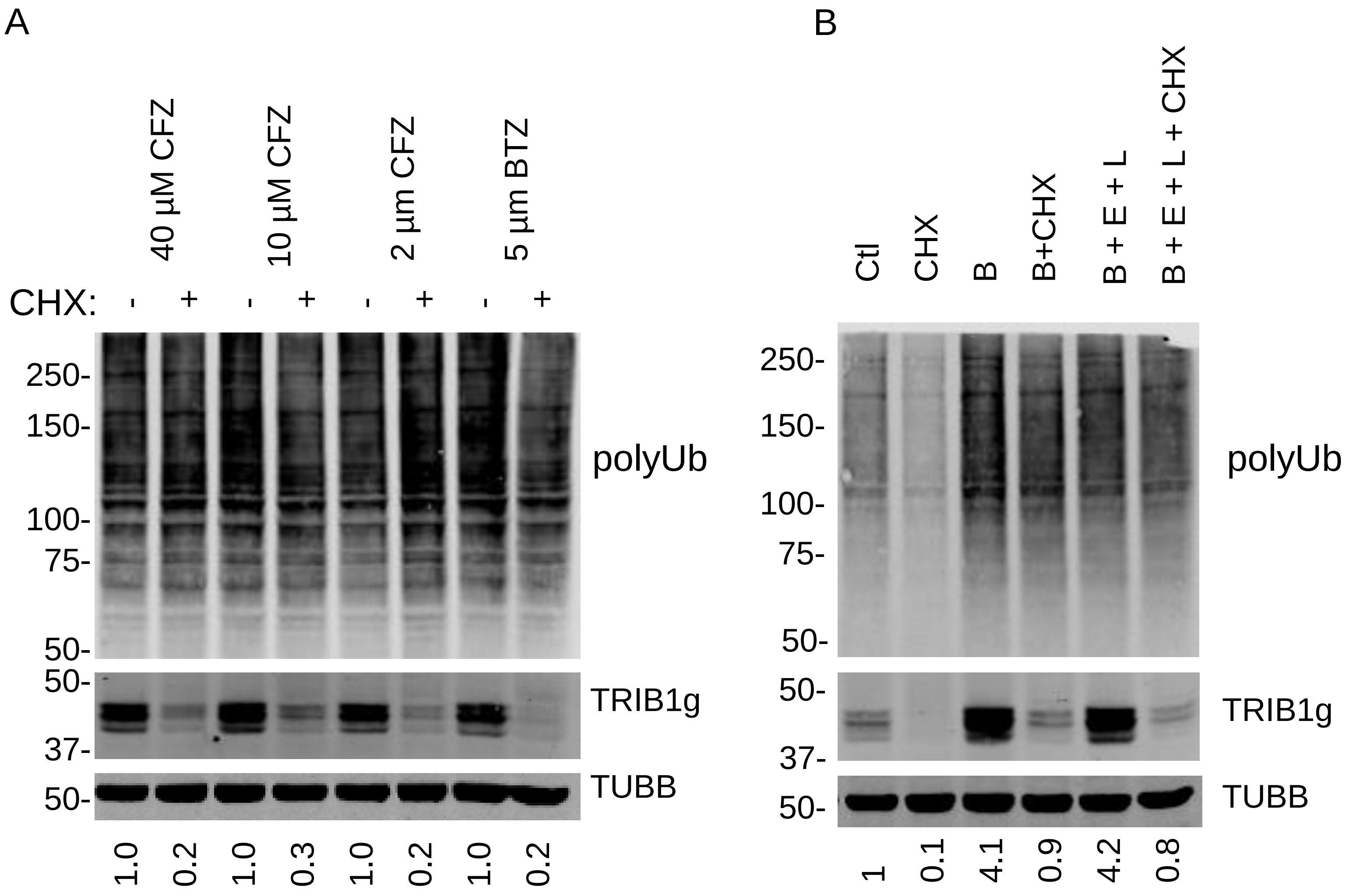

Figure S8. Loss of TRIB1 to CHX in HepG2-T1 cells in the presence of carfilzomib or a proteasome inhibitor cocktail. Western blot analyses of HepG2-T1 cells treated with proteasome inhibitors. (A) HepG2-T1 cells were treated with the indicated carfilzomib, either alone or in the presence of CHX for 5 h; carfilzomib was added 5 min prior to CHX (10 µg/ml). (B) cells were treated for 5 h with a 5 min preincubation with BTZ (B), vehicle (Ctl, 1% DMSO) or a combination of BTZ (B, 5 µM), Lactacystin (L, 10 µm) and Epoxomicin (E, 2 µm) and/or with 10 µg/ml cycloheximide (CHX), where indicated. Experiments were repeated twice with similar results. Quantifications are the TUBB-corrected TRIB1 signal and are normalized to the Ctl value (B) or the matching CHX-free value (A). Experiments were repeated twice, with similar results

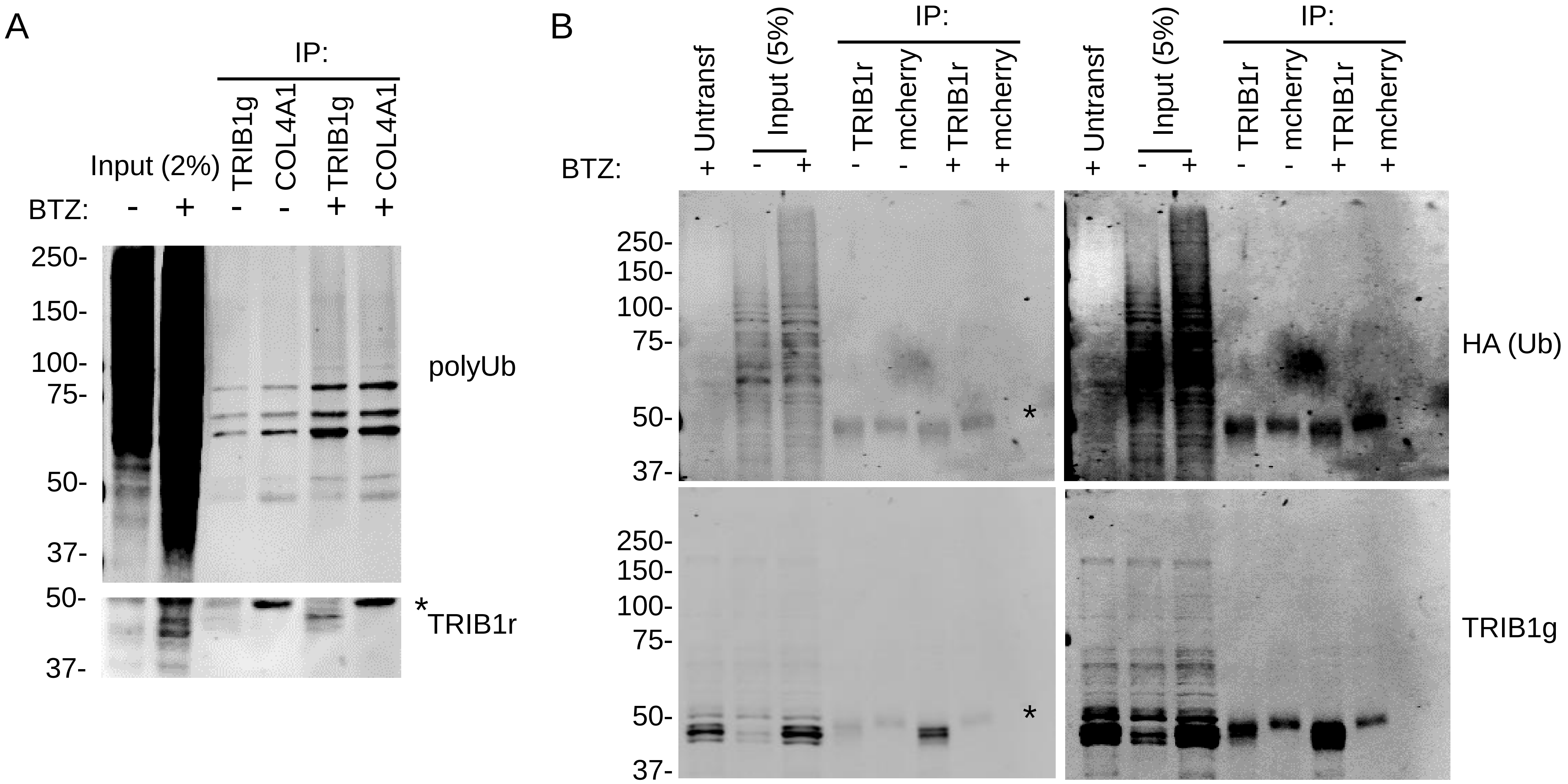

Figure S9. Immunoprecipitated TRIB1 shows no evidence of ubiquitylation. HepG2-T1 cells, treated with either BTZ (+, 5 h, 5 µM) or vehicle (-, 0.25% DMSO) for 5 h were immunoprecipitated under native (A) or denatured (B) conditions. (A) immunoprecipitation with either TRIB1g or COL4A1 goat polyclonal antibodies and analyzed by Western blotting using rabbit anti-Ub (top) or rabbit anti-TRIB1 (bottom). Detection was sequential (Ub then TRIB1r). (B) HepG2-T1 cells were transfected with a construct expressing hemagglutinin tagged ubiquitin for 48 h. An untransfected, BTZ treated control is also shown (Untransf). Denatured lysates were subjected to immunoprecipitation with anti-TRIB1 (TRIB1r) or control (mCherry) rabbit antibodies. Detection was performed with mouse anti-HA and goat anti-TRIB1 antibodies, as indicated. Two different exposures are shown. Experiments were repeated twice, with similar results. * indicates a non-specific (IgG heavy chain) band.

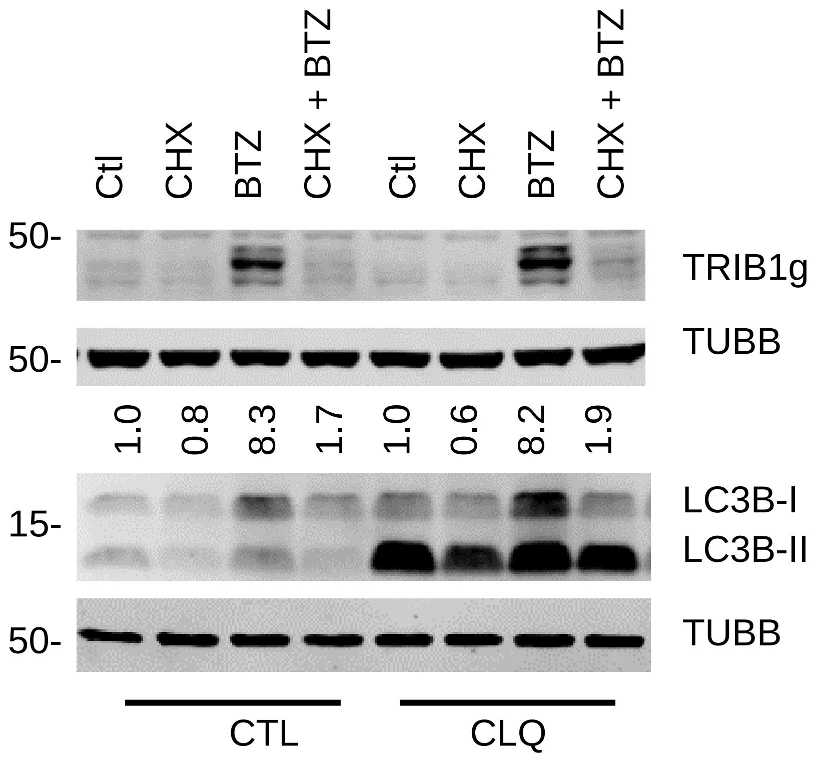

Figure S10. Autophagy inhibition does not stabilize TRIB1. Western blot analysis of HepG2-T1 cells incubated for 24 h in the presence of CLQ (5 µM) to block autophagy. BTZ and CHX were added during the last 5 h of incubation. Samples were resolved on 8% and 15% gels for TRIB1g and LC3B analysis, respectively. Experiment was repeated twice with similar results.

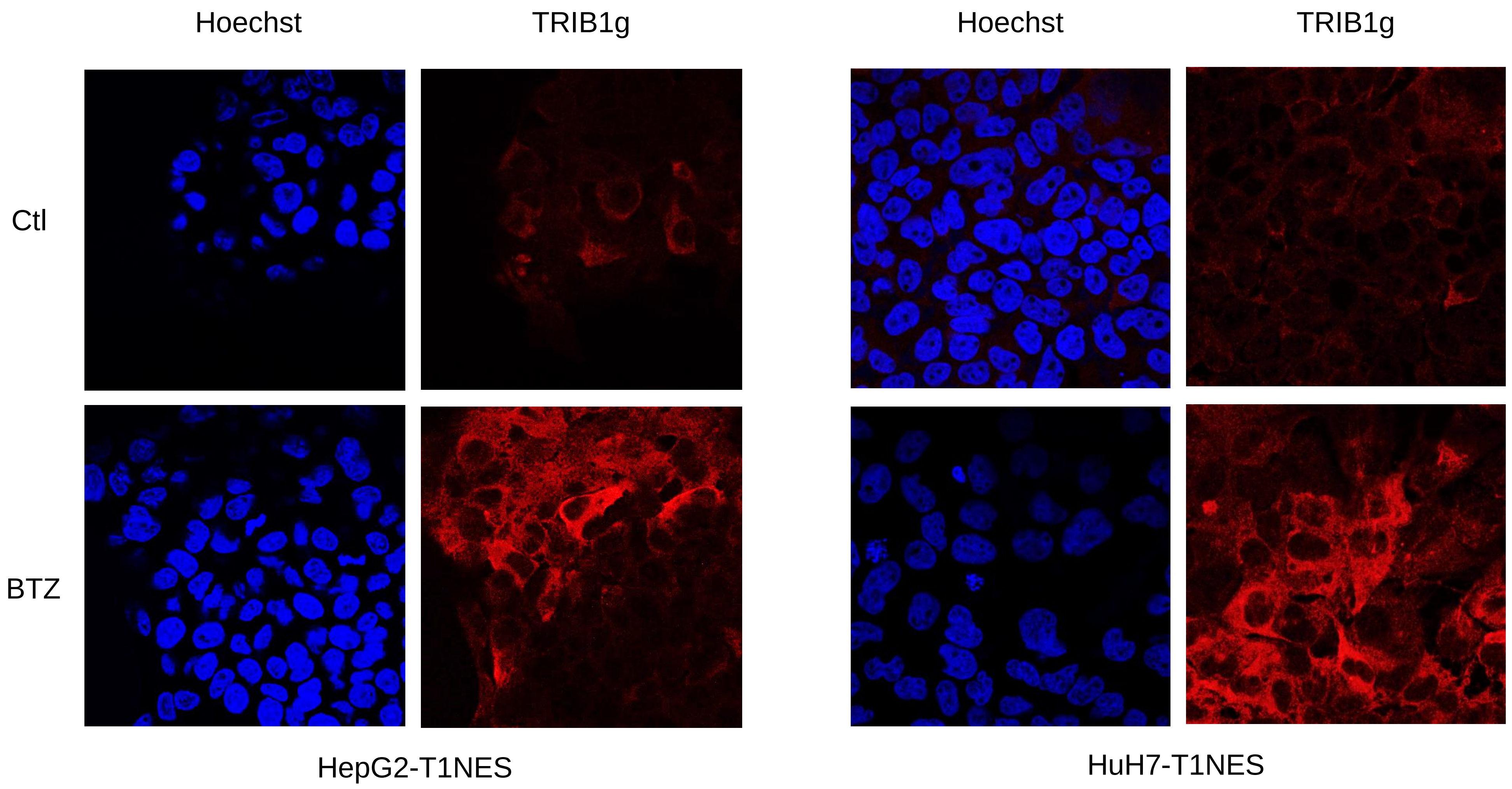

Figure S11. TRIB1NES expression in transduced HuH-7 and HepG2. HuH-7 and HepG2 cells stably transduced with TRIB1NES were treated for 5 h with BTZ. Fixed cells were permeabilized and incubated for 16 h with TRIB1g antibody. TRIB1g was detected with an Alexa 633 donkey anti-goat Ab. Nuclei were counterstained with Hoechst. Experiment was repeated twice with similar results

Supplementary Materials and Methods

Antibodies

TRIB1 (goat) : GeneTex (GTX88755)

TRIB1 (rabbit, Western blot): MilliporeSigma (09-126)

Note that lots were found to be quite variable in quality, particularly for rabbit raised TRIB1 antibodies.

TRIB1 (rabbit, denaturing IP): GenTex (GTX113459)

TUBB: GeneTex (GTX11307)

Ubiquitin: Cell Signaling (#43124)

LC3B: Cell Signaling (#3868)

Chemicals:

Actinomycin D: Cell Signaling #15021

Bortezomib: Cayman Chemical #10008822

Lactacystin: Cayman Chemical #70980

Epoxomycin: Cayman Chemical #10007806

Carfilzomib: Selleckchem S2853

PD98059: Cell Signaling #9900

SB203580: Cell Signaling #5633

SP600125: Cell Signaling #8177

Chloroquine: Cell Signaling #14774

Cycloheximide: Sigma C-7698

Tunicamycin: Cell Signaling #12819

siRNA:

from ThermoFisher (silencer select):

PSMB3si : s11348

Negative Control No. 1 siRNA: 4390844

TRIB1: s19942

ATF3: s1699

RT-qPCR primers (Forward, Reverse)

BiP and CHOP were from

https://pubmed.ncbi.nlm.nih.gov/28630443/

PPIA

ACCGTGTTCTTCGACATTGC
TTCTGTGAAAGCAGGAACCC

TRIB1

TTCAAGCAGATTGTCTCCG
CATCCACACTGGACGCGAG

BiP
ACC ACC TAC TCC TGC GTC
TTG GAG GTG AGC TGG TTC T

CHOP
CCT GGA AAT GAA GAG GAA GAA TC
ACT GGA ATC TGG AGA GTG AGG

TRIB1 constructs amino acid sequences:

TRIB1NES
MRVGPVRSAMSGASQPRGPALLFPATRGVPDLALKLAGLDIECSSPPDYLSPPGSPCSPQPPPAAPGAGGGSGSAPGP
SRIADYLLLPLAEREHVSRALCIHTGRELRCKVFPIKHYQDKIRPYIQLPSHSNITGIVEVILGETKAYVFFEKDFGDMHSYVR
SRKRLREEEAARLFKQIVSAVAHCHQSAIVLGDLKLRKFVFSTEERTQLRLESLEDTHIMKGEDDALSDKHGCPAYVSPEIL
NTTGTYSGKAADVWSLGVMLYTLLVGRYPFHDSDPSALFSKIRRGQFCIPEHISPKARCLIRSLLRREPSERLTAPEILLHP
WFESVLEPGYIDSEIGTSDQIVPEYQEDSDISSFFC

TRIB1dPEST
MRVGPVRSAMSGASQPRGPALLFPATRGVPAKRLLDADDAAAVAAKCPRLSEERDEGDKWRNKKFELGLEERDEGDK
WRNKKFELGGPSRIADYLLLPLAEREHVSRALCIHTGRELRCKVFPIKHYQDKIRPYIQLPSHSNITGIVEVILGETKAYVFFE
KDFGDMHSYVRSRKRLREEEAARLFKQIVSAVAHCHQSAIVLGDLKLRKFVFSTEERTQLRLESLEDTHIMKGEDDALSD
KHGCPAYVSPEILNTTGTYSGKAADVWSLGVMLYTLLVGRYPFHDSDPSALFSKIRRGQFCIPEHISPKARCLIRSLLRREP
SERLTAPEILLHPWFESVLEPGYIDSEIGTSDQIVPEYQEDSDISSFFCGSRDSR

TRIB1Δ2-51
MDIECSSPPDYLSPPGSPCSPQPPPAAPGAGGGSGSAPGPSRIADYLLLPLAEREHVSRALCIHTGRELRCKVFPIKHYQD
KIRPYIQLPSHSNITGIVEVILGETKAYVFFEKDFGDMHSYVRSRKRLREEEAARLFKQIVSAVAHCHQSAIVLGDLKLRKFV
FSTEERTQLRLESLEDTHIMKGEDDALSDKHGCPAYVSPEILNTTGTYSGKAADVWSLGVMLYTLLVGRYPFHDSDPSAL
FSKIRRGQFCIPEHISPKARCLIRSLLRREPSERLTAPEILLHPWFESVLEPGYIDSEIGTSDQIVPEYQEDSDISSFFC

TRIB1Δ2-91
MDIADYLLLPLAEREHVSRALCIHTGRELRCKVFPIKHYQDKIRPYIQLPSHSNITGIVEVILGETKAYVFFEKDFGDMHSYV
RSRKRLREEEAARLFKQIVSAVAHCHQSAIVLGDLKLRKFVFSTEERTQLRLESLEDTHIMKGEDDALSDKHGCPAYVSPEI
LNTTGTYSGKAADVWSLGVMLYTLLVGRYPFHDSDPSALFSKIRRGQFCIPEHISPKARCLIRSLLRREPSERLTAPEILLHP
WFESVLEPGYIDSEIGTSDQIVPEYQEDSDISSFFC
